# Silicanin-1 is a conserved diatom membrane protein involved in silica biomineralization

**DOI:** 10.1186/s12915-017-0400-8

**Published:** 2017-07-24

**Authors:** Alexander Kotzsch, Philip Gröger, Damian Pawolski, Paul H. H. Bomans, Nico A. J. M. Sommerdijk, Michael Schlierf, Nils Kröger

**Affiliations:** 1 B CUBE Center for Molecular Bioengineering, CMCB, TU Dresden, Arnoldstrasse 18, 01307 Dresden, Germany; 2 Department of Chemical Engineering and Chemistry, Laboratory of Materials and Interface Chemistry & Center of Multiscale Electron Microscopy, P.O. Box 513, 5600 MB Eindhoven, The Netherlands; 3 Institute for Complex Molecular Systems, Eindhoven University of Technology, P.O. Box 513, 5600 MB Eindhoven, The Netherlands; 4 Department of Chemistry and Food Chemistry, TU Dresden, 01062 Dresden, Germany

**Keywords:** Diatom biosilica, Biomineralization vesicles, Transmembrane protein, Vesicle biogenesis, Exocytosis, Time-lapse confocal fluorescence microscopy, Protein self-assembly, Cryo-TEM, Silica formation activity

## Abstract

**Background:**

Biological mineral formation (biomineralization) proceeds in specialized compartments often bounded by a lipid bilayer membrane. Currently, the role of membranes in biomineralization is hardly understood.

**Results:**

Investigating biomineralization of SiO_2_ (silica) in diatoms we identified Silicanin-1 (Sin1) as a conserved diatom membrane protein present in silica deposition vesicles (SDVs) of *Thalassiosira pseudonana*. Fluorescence microscopy of GFP-tagged Sin1 enabled, for the first time, to follow the intracellular locations of a biomineralization protein during silica biogenesis in vivo. The analysis revealed incorporation of the N-terminal domain of Sin1 into the biosilica via association with the organic matrix inside the SDVs. In vitro experiments showed that the recombinant N-terminal domain of Sin1 undergoes pH-triggered assembly into large clusters, and promotes silica formation by synergistic interaction with long-chain polyamines.

**Conclusions:**

Sin1 is the first identified SDV transmembrane protein, and is highly conserved throughout the diatom realm, which suggests a fundamental role in the biomineralization of diatom silica. Through interaction with long-chain polyamines, Sin1 could serve as a molecular link by which the SDV membrane exerts control on the assembly of biosilica-forming organic matrices in the SDV lumen.

**Electronic supplementary material:**

The online version of this article (doi:10.1186/s12915-017-0400-8) contains supplementary material, which is available to authorized users.

## Background

Diatoms are unicellular photosynthetic eukaryotes that produce cell walls made of amorphous, hydrated SiO_2_ (silica) and associated macromolecules [1, 2]. The diatom cell wall is located extracellular to the plasma membrane and completely encases the protoplast. Like in many other organisms (e.g., radiolaria, coccolithophores, sponges, foraminifers), the biomineral building blocks of diatoms are produced inside the cell in specialized vesicles and subsequently exocytosed and incorporated into the cell wall [3–5]. Diatom cell walls are composed of two different types of nanopatterned porous biosilica building blocks termed girdle bands (ring-shaped silica) and valves (complex shaped silica often with plate-, bowl-, or dome-like structure). During biosilica formation, silica deposition vesicles (SDVs) are positioned at the cytosolic site of the plasma membrane precisely opposite the cell wall region where a new biomineral building block will be integrated. The site-specific assembly of SDVs and exocytosis of the biosilica building blocks is a striking example of polarized intracellular membrane trafficking [6]. Several components involved in silica formation have previously been identified (see below), but the molecular machineries for SDV biogenesis and exocytosis have so far remained unknown [6–8]. The SDVs are closely associated with the actin filaments and microtubules which likely play a role in positioning and shaping of the SDVs [1, 7, 9–11]. It has been suggested that the cytoskeleton may guide morphogenesis of the porous silica nanopatterns through interactions with proteins that span the SDV membrane. Such membrane proteins would carry cytoskeleton binding domains on the cytosolic side and mineral interaction domains on the part of the protein that is exposed to the lumen of the SDV [12]. To date, there are no published reports on SDV membrane proteins from diatoms or any other silica forming organisms. In fact, hardly any information is available about the membrane proteins of eukaryotic biomineralization vesicles due to the lack of methods for isolating these subcellular compartments.

Previous biochemical analysis has led to the identification of unique proteins (silaffins, cingulins, silacidins) and long-chain polyamines (LCPA) as organic components of diatom biosilica. Most of the biosilica-associated proteins are highly charged and hydrophilic, predicted to be intrinsically disordered, and some of them have been shown to highly accelerate silica formation from monosilicic acid solutions in vitro [13–17]. A recent proteomics analysis revealed several novel biosilica-associated proteins with unknown functions in the diatom *Thalassiosira pseudonana* [17]. One of these, SiMat7, differs markedly from silaffins, cingulins, and silacidins regarding amino acid composition and predicted secondary structure. In the present work, we have investigated the function of SiMat7 by (1) determining its intracellular locations at different stages of the cell cycle, (2) probing its association with cellular membranes and with biosilica, and (3) analyzing its self-assembly properties and silica formation activity. Here, we demonstrate that SiMat7 is the founding member of a novel family of silica biomineralization proteins, which we named silicanins. Accordingly, SiMat7 was re-named silicanin-1 (abbreviated Sin1).

## Results

### Molecular architecture and sequence conservation of Sin1

**Fig. 1:**
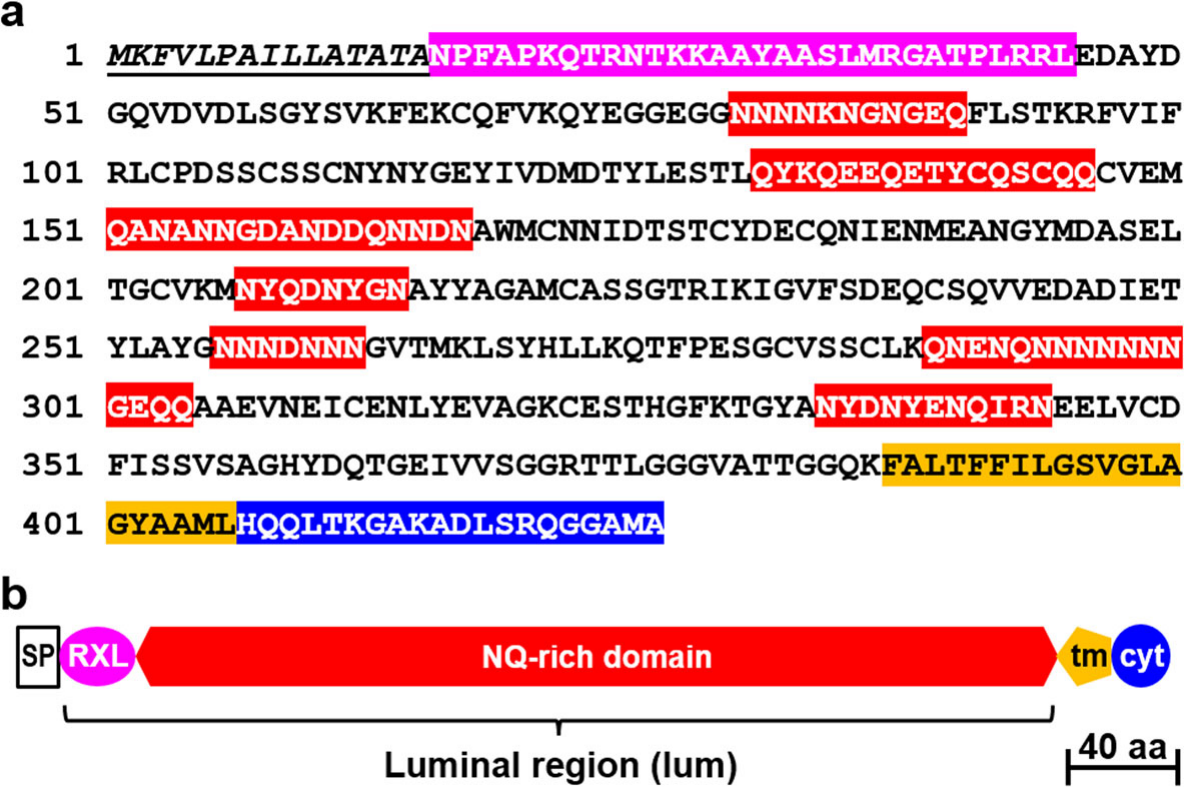
Sequence analysis of Sin1. **a** Analysis of the amino acid sequence of Sin1. The signal peptide is depicted in italics and underlined, the RXL domain is highlighted in purple, and clusters that are rich in N and Q are presented on a red background. The transmembrane helix is highlighted in *orange* and the cytosolic domain in *blue*. The N-terminal signal peptide and the transmembrane helix were identified using the SignalP v.4.1 [47] and TMHMM v.2 [48] webservers, respectively. **b** Schematic of the domain arrangement in Sin1. *SP* signal peptide, *RXL* RXL domain, *tm* transmembrane helix, *cyt* cytosolic domain

Sin1 is comprised of 426 amino acids and is a predicted type 1 transmembrane protein with a 20 amino acid cytosolic domain at the C-terminus preceded by a single transmembrane helix of 20 amino acids (Fig. 1). The cytosolic domain does not contain known cytoskeleton binding sites or any other known motifs. The remaining part of Sin1 is predicted to be exposed to the extracellular space or the lumen of a secretory compartment due to the presence of an N-terminal signal peptide for co-translational import into the endoplasmic reticulum (Fig. 1). The signal peptide is followed by a stretch of 30 amino acids ending with the tripeptide RRL, which is typical for many diatom biosilica-associated proteins and is denoted the RXL domain (Fig. 1) [14–17]. The majority of Sin1 is composed of a 341 amino acid polypeptide region rich in asparagine and glutamine, which are often present in clusters (NQ-rich domain). The NQ-rich domain of Sin1 also contains 18 cysteine residues, and secondary structure analysis predicts it to be folded with 28% α helix, 14% β sheet, and 58% disordered regions. This suggests that the 3D structure of Sin1 is very different from those of silaffins, cingulins, and silacidins, which contain only one or no cysteine residues and are predicted to adopt entirely random coil structures. Sin1 does not exhibit significant sequence similarity to any other previously described silica-associated proteins. The genome of *T. pseudonana* encodes a protein with 55% sequence identity to Sin1, which we coined Sin2 (Additional file 1: Figure S1).

A search in the NCBI database retrieved homologous proteins exclusively from diatoms (note, only proteins with an E-value lower than 1 × 10^–50^ were considered as homologs). We then extended our search for Sin1 homologs by performing a Basic Local Alignment Search Tool (BLAST) search in the Microbial Eukaryote Transcriptome Sequencing Project (MMETSP) database, which contains a large amount of gene sequences of eukaryotic microbes that are absent from the NCBI database [18]. This retrieved Sin1 homologs from 70 diatom species and from two non-diatom organisms (Additional file 1: Table S1). The three closest Sin1 homologs from centric diatoms have a higher sequence identity to Sin1 (66%) than the three closest homologs from pennate diatoms (46–47%) (Additional file 1: Table S2). The two non-diatom organisms harboring Sin1 homologs are the amoeboid alga *Rhizochromulina marina* (62% amino acid sequence identity) and the colepid ciliate *Tiarina fusa* (49% sequence identity). Sin1 homologs appeared to be absent from other unicellular silica-forming organisms in the MMETSP database such as synurophyceae (four species in the MMETSP database), chrysophyceae (six species), dictyochophyceae except for *Rhizochromulina marina* (eight species), and loricate choanoflagellates (one species). Unfortunately, genome or transcriptome data from other biosilica-forming organisms such as actinophryids, radiolarians and the silica-forming coccolithophore *Prymnesium neolepis* [19] were not publicly available. Furthermore, Sin1 homologs could not be found in the genome of the siliceous sponge demosponge *Amphimedon queenslandica* [20].

All Sin1 homologues identified here are also predicted type 1 transmembrane proteins, share the same domain composition and arrangement, and the positions of the 18 cysteine residues in their NQ domains are conserved (Additional file 1: Figure S1). Therefore, we regard these proteins as members of the silicanin protein family, and assume that they exhibit 3D structures and biological functions that are very similar to Sin1.

### Membrane association of Sin1

**Fig. 2:**
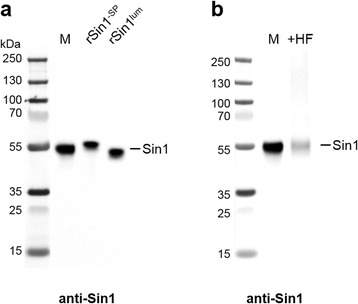
Western blot analysis using anti-Sin1 antibodies. *M* total membrane fraction from *T. pseudonana*; *M + HF* total membrane fraction from *T. pseudonana* after treatment with anhydrous HF; **a** Apparent molecular mass of native Sin1 in comparison to recombinant proteins rSin1^-SP^ and rSin1^lum^ (Additional file 1: Figure S10). **b** Effect of anhydrous HF on the apparent molecular mass of native Sin1. The left lanes in **a **and **b** contain molecular mass standard proteins﻿

To examine whether Sin1 is membrane-associated as predicted (see above), we isolated total membranes from *T. pseudonana* according to an established protocol [21]. Using Western blot analysis, the membranes were probed for the presence of Sin1 with an antibody directed against the luminal region of Sin1 (i.e., the combined RXL and NQ domains; Fig. 1b). A single intense band of 55 kDa apparent molecular mass was detected, which is about 10 kDa higher than was expected for a Sin1 molecule lacking the signal peptide (predicted molecular mass: 45.2 kDa; Fig. 2a). To investigate whether the difference in apparent molecular mass was caused by an unusual migration behavior of Sin1 on sodium dodecyl sulfate (SDS)-polyacrylamide gel electrophoresis (PAGE), we expressed two recombinant Sin1 proteins in *E. coli*. Protein rSin1^-SP^ (molecular mass: 45.1 kDa, Additional file 1: Figure S2a) contained all Sin1 domains except the signal peptide, and protein rSin1^lum^ (molecular mass: 40.7 kDa) was only composed of the luminal region (Additional file 1: Figure S2b). On SDS-PAGE, both proteins also had apparent molecular masses around 55 kDa, demonstrating that Sin1 indeed exerts an aberrant migration behavior on SDS-PAGE (Fig. 2a). Therefore, we concluded that the 55 kDa band in the membrane fraction of *T. pseudonana* corresponds to Sin1. Its apparent molecular mass is slightly higher than rSin1^lum^, which would be consistent with the presence of the transmembrane helix and cytosolic domain in Sin1 provided that this protein does not contain significant amounts of post-translational modifications. After treatment of *T. pseudonana* membranes with anhydrous HF, which removes O-linked glycans and O-phosphoryl moieties [22], the apparent molecular mass of Sin1 remained unchanged (Fig. 2b). This indicates the absence of substantial amounts of glycan and phosphate moieties in native Sin1. The fact that the apparent molecular mass of Sin1 is is slightly lower than rSin1^-SP^ (Fig. 2a) suggests that Sin1 may lack the RXL domain. Proteolytic removal of RXL domains by a yet unknown protease has been observed in other biosilica-associated proteins such as silaffins [13].

To test whether the predicted transmembrane domain of Sin1 is integrated into the lipid bilayer, isolated total membranes of *T. pseudonana* were extracted with a carbonate containing buffer at pH 11.5. Under these conditions, proteins containing a domain that fully penetrates the lipid bilayer remain bound to the membrane, whereas proteins that are peripherally associated with the membrane become completely solubilized [23]. Western blot analysis revealed that approximately half of the Sin1 molecules remained associated with the carbonate extracted membranes (Additional file 1: Figure S3a, Additional file 1: Table S3). Under the same conditions, the peripheral membrane protein AtpB (β-subunit of plastidic/mitochondrial ATP synthase) was completely extracted from the membranes, and the integral membrane protein PsbD (5 transmembrane helices) remained entirely in the membrane (Additional file 1: Figure S3b, c, and Table S3). Regarding the partial extractability from membranes using alkaline carbonate buffer, Sin1 is similar to other type 1 transmembrane proteins, for example, like the β subunit of the SRP receptor [24] and the lysosomal transmembrane protein NCU-G1 [25]. Therefore, the fact that approximately half of the Sin1 protein molecules remain associated with the membrane rather than becoming fully extracted by alkaline carbonate buffer is regarded as a proof for transmembrane anchoring of Sin1.

### Localization and silica embedment of Sin1

**Fig. 3:**
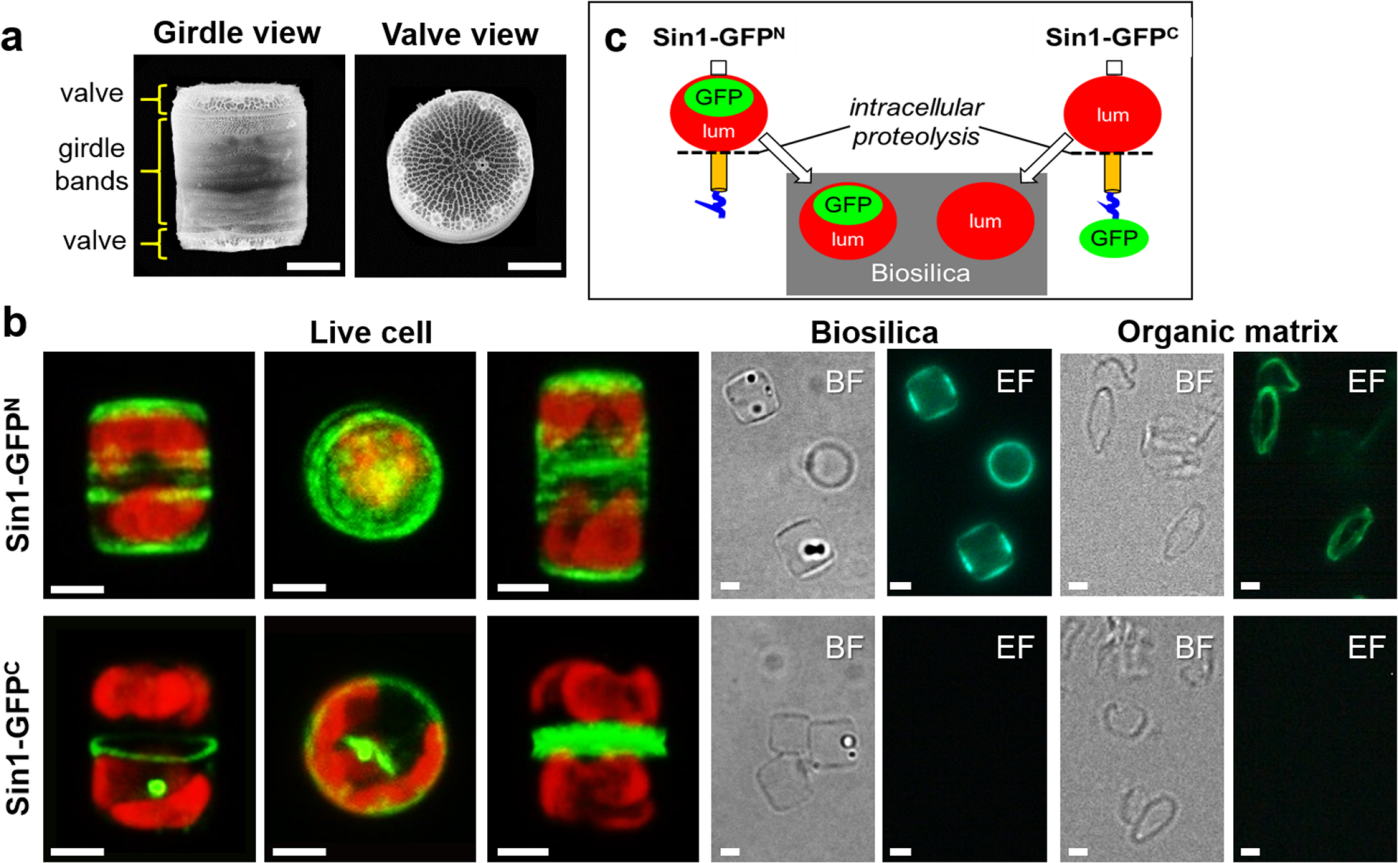
Localization of Sin1-GFP fusion proteins in *T. pseudonana*. **a** SEM images of biosilica from individual cells in two different orientations. **b** Live cells, biosilica, and biosilica-associated organic matrix from transformant strains expressing Sin1-GFP^N^ or Sin1-GFP^C^. The fusion proteins were expressed under control of the endogenous Sin1 promoter and terminator sequences. The ‘Live cell’ panels show confocal fluorescence images (z-projection) of individual cells in girdle view (*left panel*, and third panel from the *left*) and in valve view (second panel from the *left*). *Green* color indicates the GFP fusion proteins and the *red* color is caused by chlorophyll autofluorescence. The biosilica and organic matrix panels show bright field microscopy images (BF) and the corresponding epifluorescence microscopy images (EF) of material isolated from Sin1-GFP^N^- or Sin1-GFP^C^-expressing transformants. Scale bars for all images: 2 μm. **c** Proposed intracellular proteolytic processing of Sin1. Sin1 becomes cleaved by a protease between the luminal domain (lum) and the transmembrane helix (*orange*). The luminal domain is incorporated into the biosilica, while the transmembrane helix and the cytosolic domain (*blue* squiggle) become degraded

To investigate the intracellular location and silica association of Sin1, two GFP fusion proteins, Sin1-GFP^N^ and Sin1-GFP^C^, were independently expressed in *T. pseudonana*. In Sin1-GFP^N^ the GFP is located right between the RXL domain and the NQ-rich domain (i.e., in the predicted extracellular/luminal region of Sin1). In Sin1-GFP^C^, the GFP-tag is attached to the end of the predicted cytosolic domain. Fluorescence microscopy analysis of transformant cells expressing Sin1-GFP^N^ revealed GFP fluorescence in the valve and girdle band regions of live cells and in isolated biosilica (Fig. 3a, b top panel). After complete removal of the silica, the GFP was present in ring-shaped, purely organic structures (Fig. 3b top panel ). This confirmed that Sin1 is a component of the previously described biosilica-associated insoluble organic matrices [16, 17] from which it has recently been identified by proteomics analysis [17]. Accessibility experiments using anti-Sin1 antibodies, which were directed against the luminal domain of Sin1 (amino acids 25–383), demonstrated that less than 20% of the Sin1 molecules were accessible in biosilica isolated from Sin1-GFP^N^ expressing cells compared to their accessibility in the silica-free insoluble organic matrices (Additional file 1: Figure S4, Additional file 1: Table S4). This result indicated that the Sin1 molecules are largely embedded inside the biosilica, which implies that they are exposed to the lumen of the SDVs during silica deposition in vivo.

In cells expressing Sin1-GFP^C^, GFP-fluorescent ring-like structures and plate-like structures appeared transiently during girdle band and valve morphogenesis, respectively (Fig. 3a, b, bottom panel). Strong GFP fluorescence was also associated with intracellular spherical structures that were moving throughout the cytoplasm, whereas GFP fluorescence appeared to be absent from the biosilica cell walls (Fig. 3b bottom panel, Additional file 2: Movie S1). Indeed, biosilica and organic matrices prepared from cells expressing Sin1-GFP^C^ did not exhibit any GFP fluorescence (Fig. 3b, bottom panel). This seemed to indicate that Sin1 is absent from the biosilica and the organic matrices, which would contradict the result obtained with cells expressing Sin1-GFP^N^. To resolve this discrepancy, *T. pseudonana* transformants were generated to express the fusion protein Sin1-mT2^N^-Venus^C^. In this fusion protein the cyan fluorescing protein mTurquoise2 (mT2) was positioned within the luminal region (between the RXL domain and NQ domain) and the yellow fluorescing protein Venus was attached to the C-terminus. Live transformant cells exhibited both cyan fluorescence and yellow fluorescence (Additional file 1: Figure S5a), confirming that the full length double fluorescent-tagged Sin1 protein molecules were expressed. The cyan fluorescence was present in the biosilica of live cells (Additional file 1: Figure S5a), in isolated biosilica, and in the insoluble organic matrices (Additional file 1: Figure S5b). In contrast, the yellow fluorescence was absent from the biosilica and the insoluble organic matrices (Additional file 1: Figure S5a, b). This result can be explained by assuming a proteolytic cleavage between the luminal region and the C-terminal part of Sin1 during silica biogenesis. Only the luminal domain of Sin1 rather than the transmembrane helix and cytosolic domain would become incorporated into the biosilica (Fig. 3c). This scenario is also consistent with the presence and absence of GFP fluorescence in biosilica from transformants expressing Sin1-GFP^N^ and Sin1-GFP^C^, respectively (﻿see ﻿Fig. 3b). The heptapeptide motif GGQKFAL, which is right at the transition of the luminal region to the transmembrane domain, is perfectly conserved in all silicanin sequences (see Additional file 1: Figure S1) and might be the recognition site for a silicanin-specific protease. To exclude the possibility that the lack of GFP fluorescence in biosilica from Sin1-GFP^C^ transformants was due to denaturation of GFP, immunolabeling experiments were performed using a polyclonal anti-GFP primary antibody and an AlexaFluor647 (AF) conjugated secondary antibody. The GFP fluorescent biosilica and organic matrices from Sin1-GFP^N^ expressing cells also exhibited AF fluorescence, demonstrating accessibility of biosilica and organic matrix bound GFP molecules for the anti-GFP antibodies (Additional file 1: Figure S6). No AF fluorescence was detected in the biosilica and the organic matrices from Sin1-GFP^C^ cells (Additional file 1: Figure S6), thereby confirming the absence of GFP, which is in agreement with C-terminal proteolytic processing of Sin1 upon incorporation into the biosilica (Fig. 3).

### Time-lapse imaging of Sin1-GFP^C^

**Fig. 4:**
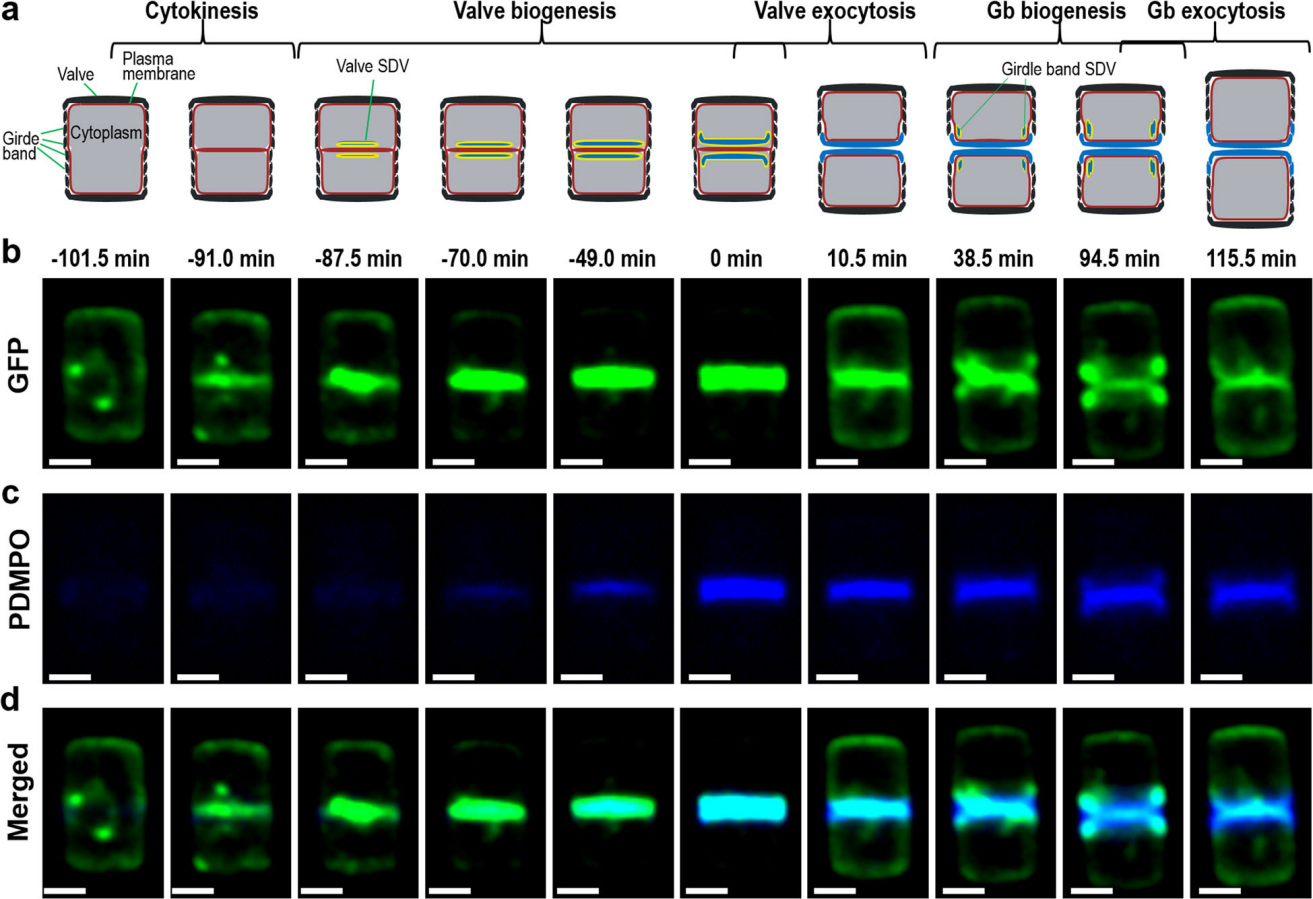
Localization of Sin1-GFP^C^ around the time of valve biogenesis. **a** Schematic drawings illustrating the different stages of the diatom cell cycle. For simplicity, intracellular compartments, except for the SDVs, have been omitted. *Black* and *blue* colors indicate mature biosilica and newly produced biosilica, respectively. *Red* and *yellow* colors depict the plasma and SDV membranes, respectively. **b–d** Selected images from time-lapse confocal fluorescence microscopy of Sin1-GFP^C^ labeled with PDMPO are shown (Additional file 2: Movie S1). The time above the images relates to the peak of the GFP and PDMPO fluorescence (Fig. 5), which is set as t = 0 min. Panel **b** shows the GFP fluorescence (*green*), panel **c** the PDMPO fluorescence (*blue*), and panel **d** an overlay of GFP and PDMPO fluorescence (note: a superposition of *green* and *blue* fluorescence appears *cyan*). All images are projections of nine z-planes. Scale bars: 2 μm

To investigate the location of Sin1 during the cell cycle, time lapse confocal fluorescence microscopy with individual cells expressing Sin1-GFP^C^ was performed. Biosilica produced during imaging was labeled by pre-loading the cells with the dye 2-(4-pyridyl)-5-((4-(2-dimethylaminoethylaminocarbamoyl)methoxy)phenyl)oxazole (PDMPO). PDMPO is known to accumulate in silica deposition vesicles and remains permanently entrapped inside the biosilica also after exocytosis, but it does not stain mature biosilica that is already present on the cell surface [26]. Inside Sin1-GFP^C^ expressing cells, several GFP-fluorescent spherical particles were present. The particles were quite mobile but most of the time remained close to the region where the cleavage furrow will appear (i.e., the mid-cell region), and seem to be associated with weakly GFP fluorescent mobile strands (Additional file 2: Movie S1). During the entire cell cycle, GFP fluorescence is also present throughout the plasma membrane (Additional file 2: Movie S1). In Fig. 4, still images from Additional file 2: Movie S1 from the GFP channel (Sin1-GFP^C^ localization; Fig. 4b), from the PDMPO channel (biosilica localization; Fig. 4c), and the corresponding merged images from the two channels (Fig. 4d) are shown. Additionally, schematic drawings are presented showing the characteristic stages of the cell cycle (Fig. 4a). We assume that biogenesis of valve biosilica is complete when GFP and PDMPO fluorescence in the mid-cell region reach their maximum intensity. This time point was defined as t = 0 min (approximately in the middle of Additional file 2: Movie S1). Therefore, all events preceding the completion of valve biogenesis are assigned negative times.

**Fig. 5:**
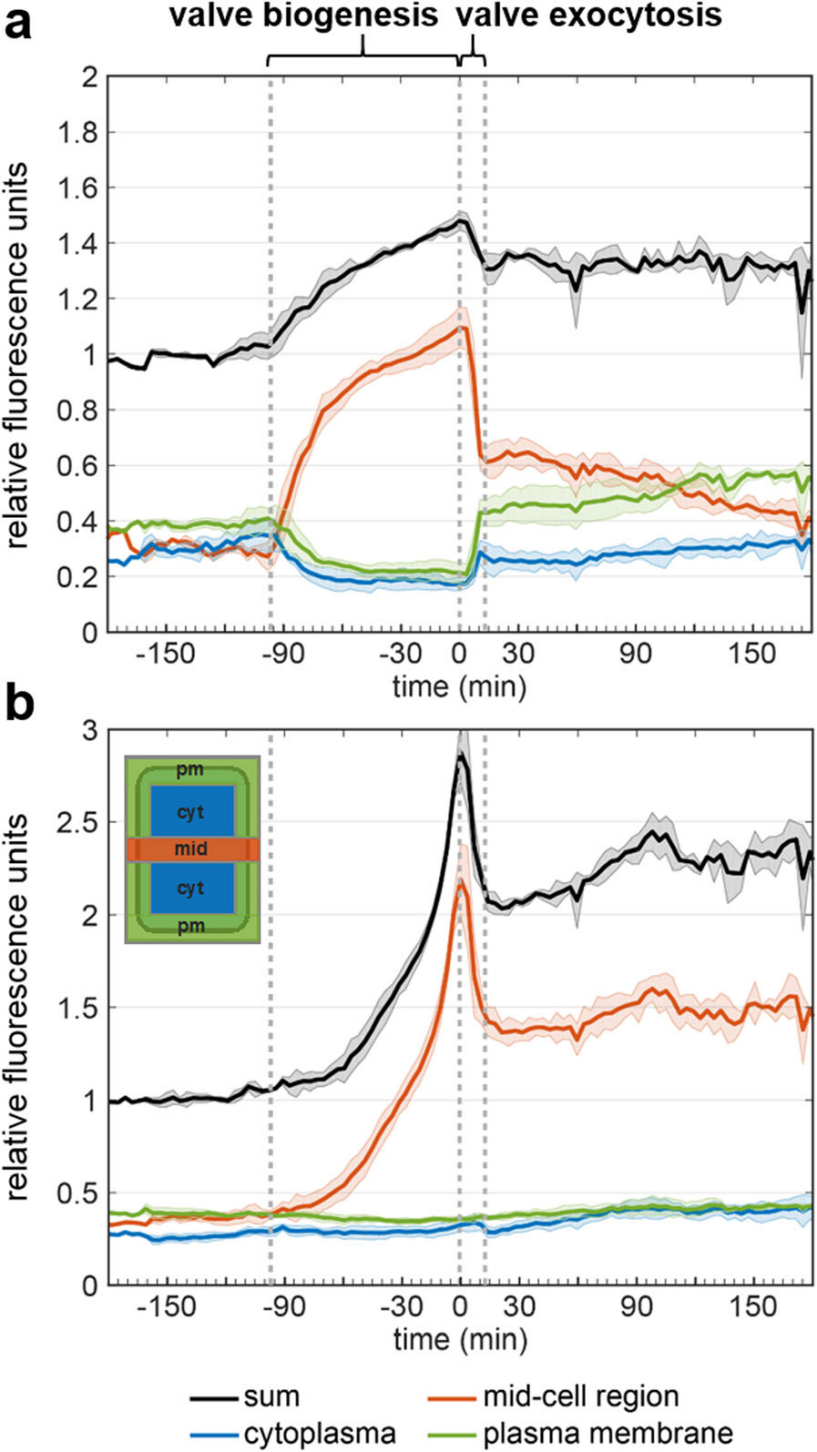
Time-dependent quantitative analysis of region-specific (**a**) GFP and (**b**) PDMPO fluorescence during valve formation in Sin1-GFP^C^ expressing cells. The plots show the averaged results from four different transformant cells labeled with PDMPO. From each cell, images were recorded in 3.5 min intervals, and the fluorescence intensities in different regions of the cell were determined. From each frame, z-projections were generated by combining all nine z-planes. The schematic shows the delineations of the cellular regions analyzed. The *coloring* of the cellular regions in the schematic corresponds to the line coloring in the graphs. The *black lines* represent the sum of the intensities from all regions of the cell. The frame with the maximum GFP and PDMPO fluorescence during the time-lapse recording was defined as t = 0 min. This allowed alignment of the time scale of the four different cells used in our analysis. The gray-shaded areas represent the standard deviation of the averaged fluorescence intensities obtained from the four cells for each region. No standard deviation is given for time periods for which only a single cell was available for averaging

At t = –101.5 min the cell was in late interphase, and GFP fluorescence was present throughout the cell periphery (i.e., the region of the plasma membrane) as well as in spherical particles and associated strands inside the cell (Fig. 4b, t = –101.5 min). Nuclear division typically was completed by t = –95.0 min (Additional file 3: Movie S2, Additional file 1: Figure S7), and shortly thereafter GFP fluorescence was present throughout the mid-cell region (Fig. 4b, t = –91.0 min). We assume that, at this time point, cytokinesis has just been completed and thus the GFP-fluorescent plasma membranes of the two sibling cells are adjacent to one another in the mid-cell region. Shortly after cytokinesis, GFP fluorescence strongly increased in the mid-cell region starting from the center (Fig. 4b, t = –87.5 min) and expanding until it spanned the entire middle plane (Fig. 4b, t = –49.0 min). Fluorescence in the mid-cell region appeared to steadily increase until the end of valve biogenesis (Fig. 4b, t = 0 min). Less than 20 min after the onset of strong GFP fluorescence in the mid-cell region and in co-localization, PDMPO fluorescence appeared and continuously increased (Fig. 4b, c, –70.0 to 0 min). These observations demonstrated (1) the development of a valve biosilica in each sibling cell during the time period from –70 to 0 min, and (2) the co-localization of Sin1 with the valve SDVs during silica biogenesis. During the following 10 minutes, GFP fluorescence intensity in the mid-cell region decreased drastically while GFP fluorescence in the entire plasma membrane region of each sibling cell increased (Fig. 4b, t = 0 to 10.5 min). This was confirmed by quantitative analysis of the fluorescence intensity, which revealed identical fast kinetics for the GFP loss in the mid-cell region and the increase of GFP fluorescence in the plasma membrane region upon exocytosis of the valves from the two sibling cells (Figs. 4b and 5a). Consistent with this assumption was the simultaneous sudden drop in fluorescence intensity of the biosilica-bound PDMPO (Figs. 4c and 5b). Upon exocytosis, the pH in the vicinity of the biosilica changes from acidic inside the SDVs [27] to near neutral on the cell surface. In this pH range, PDMPO fluorescence intensity in the recorded wavelength range (510–540 nm) decreases with increasing pH [26, 28]. Furthermore, it is possible that PDMPO molecules, which had accumulated inside the SDVs but were not entrapped inside the newly produced biosilica, rapidly diffused into the surrounding medium upon exocytosis.

Shortly after exocytosis of the valves, the distance between the centers of the two sibling cells had increased and several spherical GFP-fluorescent particles and associated GFP-fluorescent strands reappeared in the sibling cells near their contact region (Fig. 4b, t = 38.5 min). We assume that the GFP-fluorescent spherical particles and associated strands, which were also observed before the onset of valve biogenesis (Fig. 4b, t = –101.5 min), are membrane-bounded compartments that Sin1 passes through on its way to the SDV. During valve biogenesis these structures appeared to have fused with the developing valve SDVs in the mid-cell region. Between 38.5 and 101.5 min, in each sibling cell, a GFP-labeled, ring-shaped structure was present and located near the edges of the newly produced valves (Fig. 4b, t = 94.5 min). In this location the first girdle band SDV is supposed to develop in each sibling cell. Indeed, silica biogenesis was confirmed by the co-localization of GFP with PDMPO fluorescence (Fig. 4b, c, t = 94.5 min). Like with valve exocytosis, GFP fluorescence intensity at the sites of girdle band formation rapidly decreased during exocytosis while GFP fluorescence in the entire plasma membrane simultaneously increased with the exocytosis of each girdle band (Fig. 4a, t = 115.5 min; Additional file 4: Movie S3; Additional file 1: Figure S8, t = 101.5 to 112 min).

The results from the imaging of Sin1-GFP^C^ during the cell cycle are consistent with Sin1 being anchored in the SDV membrane during silica biogenesis. We regard the strong increase of Sin1-GFP^C^ fluorescence in the mid-cell region at t = –87.5 min as the onset of valve SDV development (Fig. 4b). By t = –70 min, Sin1-GFP^C^ had fully extended across the entire mid-cell region (Fig. 4b), and only at this time point did silica deposition become noticeable (Fig. 4c). This result indicates that the development of the valve SDVs precedes the deposition of silica, which is in agreement with the results from a previous ion-abrasion electron microscopy study on *T. pseudonana* [29].

The sudden decrease of GFP fluorescence in the mid-cell region of the cell and the simultaneous increase in GFP fluorescence in the plasma membrane during exocytosis of the valve (Fig. 5a, t = 0 to 10.5 min) and girdle band biosilica (Additional file 1: Figure S8, t = 100 to 115 min) can be explained by fusion of the SDV membranes with the plasma membrane. This enabled the SDV-derived Sin1-GFP^C^ molecules to diffuse across the entire plasma membrane, thus substantially decreasing in abundance at the site of biosilica exocytosis. However, the decrease of GFP fluorescence in the mid-cell region during valve exocytosis was only partially compensated by the increase of GFP fluorescence in the plasma membrane. During this time, GFP fluorescence in the cytoplasm increased only moderately (blue trace in Fig. 5a, t = 0 to 10.5 min), and thus the sum of cellular GFP fluorescence decreased (black trace in Fig. 5a, t = 0 to 10.5 min). As GFP fluorescence outside the cell did not increase, the result suggests that a fraction of the Sin1-GFP^C^ molecules was proteolytically degraded during valve exocytosis. From the normalized GFP fluorescence data (Fig. 5a) it was estimated that the total amount of Sin1-GFP^C^ molecules increased by approximately 50% during valve biogenesis (t = –87.5 to 0 min). The subsequent drop in total GFP fluorescence indicated that roughly one-third of the newly synthesized Sin1-GFP^C^ molecules were degraded during valve exocytosis (t = 0 to 10 min). This degradation-prone fraction may be formed by Sin1-GFP^C^ molecules from which the luminal region was proteolytically cleaved off and incorporated into the biosilica. The remaining GFP-tagged C-terminal part (containing the transmembrane and cytosolic domains of Sin1) may have then become rapidly degraded, thus eliminating the GFP fluorescence. In contrast, Sin1-GFP^C^ molecules that retain the luminal region during silica biogenesis may be resistant to such fast proteolysis, and after valve exocytosis would become components of the plasma membrane.

### Properties of recombinant Sin1^lum^

**Fig. 6:**
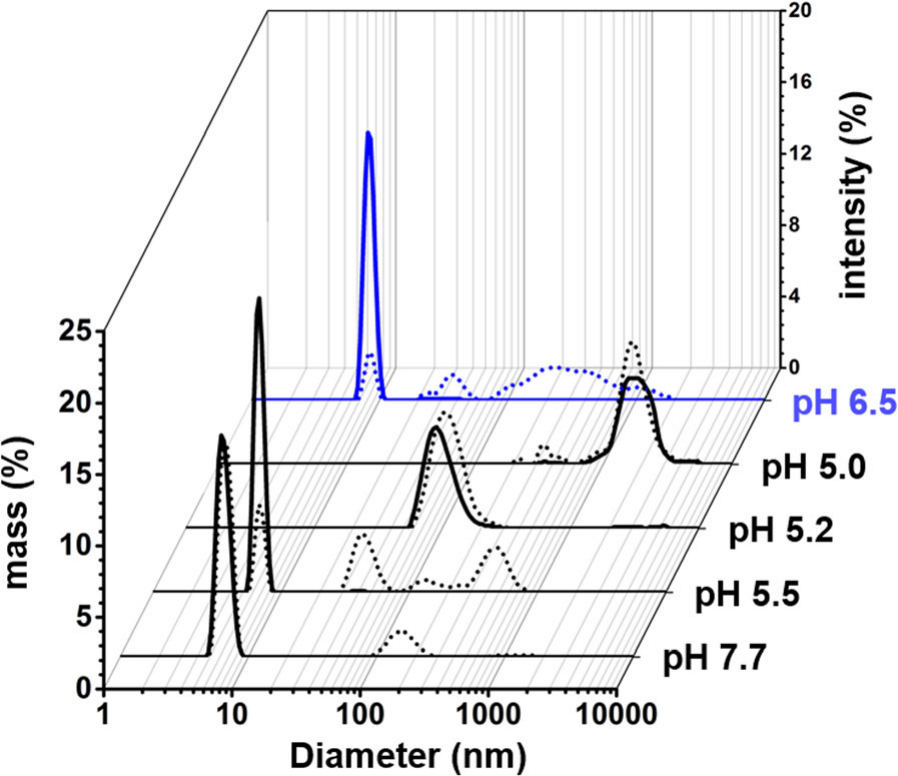
Dynamic light scattering analysis of rSin1^lum^ at different pH. A solution of rSin1^lum^ was adjusted to increasingly acidic pH values (*black traces*), and then titrated back to near neutral pH (*blue trac*e). The *black traces* show the particle distribution by mass. The *dotted lines* show the particle distribution by intensity to highlight the presence of small amounts of rSin1^lum^ clusters

**Fig. 7:**
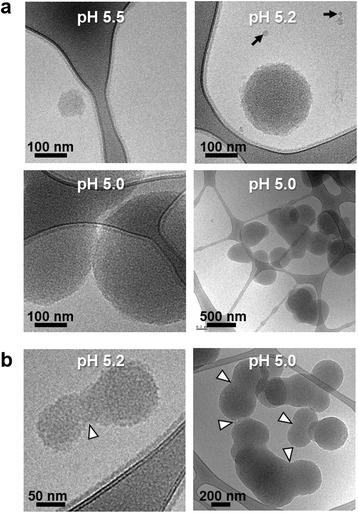
Cryo-electron microscopy analysis of rSin1^lum^ clusters. **a** Clusters at different pH values 60 min after adjustment to the indicated pH. The *black arrows* point to small clusters that consist of only 10–20 protein monomers. **b** Clusters at pH 5.2 (*left*) and pH 5.0 (*right*). The *arrowhead*s point to neck regions between two clusters that are indicative of fusion events

To further investigate the function of Sin1 in silica biogenesis, we studied the properties of the recombinantly expressed luminal region of Sin1 (rSin1^lum^, aa 25–383), which contains most of the RXL domain, the NQ domain, and a C-terminal hexahistidine tag (Additional file 1: Figures S2b, S9a). This recombinant protein comprises 84% of the Sin1 polypeptide sequence and is expected to be present at the luminal side of the SDV membrane (see above), which is a key interface in silica morphogenesis [12]. Analysis by circular dichroism spectroscopy indicated that rSin1^lum^ is a globular protein with a combined α helix and β sheet content of 40% (Additional file 1: Figure S8b, Additional file 1: Table S5). Using Ellman’s reagent, it was demonstrated that all 18 cysteine residues in rSin1^lum^ are engaged in disulfide bonds (Additional file 1: Table S6). Dynamic light scattering (DLS) revealed that rSin1^lum^ has a hydrodynamic diameter of 6.8 ± 0.6 nm at pH 7.7 (Fig. 6), which closely matches the theoretical prediction of 6.6 nm for a monomeric, globular protein with 428 amino acids (i.e., the number of amino acids in rSin1^lum^) [30]. Although the solution was almost exclusively composed of monomers (>99% by mass) at pH 7.7, a small amount of rSin1^lum^ clusters with a hydrodynamic diameter (D_h_) of 156 ± 36 nm was detected (see dotted line for pH 7.7 in Fig. 6). When the solution was acidified to pH 5.5, larger amounts of rSin1^lum^ clusters with D_h_ = 54 ± 12 nm were detected, yet the solution was still mainly composed of monomers (~ 99% by mass; Fig. 6). A further moderate increase of acidity to pH 5.2 and pH 5.0 induced quantitative formation of rSin1^lum^ clusters with D_h_ = 108 ± 36 nm and D_h_ = 1806 ± 316 nm, respectively (Fig. 6). After decreasing the acidity by adjusting the pH to 6.5, the clusters disassembled within 1 hour, yielding almost completely monomeric rSin1^lum^ (~ 98% by volume; Fig. 6). This result indicated that pH-induced assembly of rSin1^lum^ clusters is a reversible process. Cryo-electron microscopy revealed that the rSin1^lum^ clusters had spherical shapes with a relatively wide size distribution that increased with decreasing pH, and was within the size range determined by DLS (Fig. 7a). Growth of the clusters appeared to occur through fusion (Fig. 7b), but we cannot exclude the possibility that addition of rSin1^lum^ monomers also contributed to cluster growth. We assume that the pH-triggered, reversible formation of aggregates is a physiologically relevant property of Sin1, because diatom SDVs are acidic compartments [27].

As the luminal domain of Sin1 is embedded inside the silica (Additional file 1: Table S4), we investigated whether this part may be directly involved in the deposition of silica inside the SDVs. Therefore, we analyzed the silica formation activity of rSin1^lum^ in vitro at pH 5.5, which is presumed to be close to the physiological pH inside the SDVs [27, 31, 32]. Only small amounts of silica (20 ± 2 nmol SiO_2_) were formed when rSin1^lum^ was incubated with monosilicic acid. Previously, it has been shown that strongly negatively charged diatom phosphoproteins (e.g., silaffins tpSil3, tpSil1/2H, silacidins) do not have silica-formation activities by themselves, yet mixtures of the phosphoproteins with LCPA or mixtures of polyamines with phosphate exhibited high silica formation activity [14, 15, 33]. The luminal region of Sin1 is predicted to be highly negatively charged at pH 5.5 due to the presence of many aspartate and glutamate residues (14% of the total amino acids; predicted isoelectric point is 4.5). Therefore, we investigated the silica formation activity of rSin1^lum^ in the presence of LCPA. An equimolar mixture of rSin1^lum^ with LCPA (25 μM each) produced 104 ± 1 nmol SiO_2_, which was more than five- and seven-fold higher than that of rSin1^lum^ (see above) and LCPA (15 ± 5 nmol) by themselves. The silica formation activity of the rSin1^lum^-LCPA mixture had more than double the activity of an LCPA-phosphate mixture (50 ± 6 nmol SiO_2_). These data demonstrated that rSin1^lum^ interacts with LCPA, resulting in a high silica formation activity at near physiological pH conditions.

## Discussion

In the present work, we have identified Sin1 as the first SDV transmembrane protein. Bioinformatics analysis revealed that Sin1 is highly conserved throughout the diatom realm, and homologous proteins were also identified in two non-diatom organisms. One of them, the amoeboid alga *Rhizochromulina marina*, is not reported to produce biosilica, yet it belongs to the taxon *Dictyochophyceae*, which also includes silicoflagellates that form siliceous skeletons [34–36]. The other non-diatom homologue of Sin1 is present in the colepid ciliate *Tiarina fusa*, which is a protozoan that forms a shell made of calcium carbonate [37]. This suggests an evolutionary relationship between the mechanisms for the biomineralization of silica and calcium carbonate, which has recently been also demonstrated for three coccolithophore species [19]. These coccolithophore species encode silicic acid transporter-like proteins and the biomineralization of their calcium carbonate scales was perturbed by germanic acid (i.e., an inhibitor of silica metabolism) [19]. The absence of Sin1 genes in other non-diatom organisms that produce biosilica (e.g., synurophyte *Mallomonas sp.*, chrysophyte *Paraphysomonas imperforata*, silica sponge *Amphimedon queenslandica*) indicates that Sin1 is not universally required for biological silica formation.

Given the high degree of sequence similarity among Sin1 homologues in centric and pennate diatoms, we assume that Sin1 may have a fundamental role in the biogenesis of diatom biosilica, which will be discussed below. Based on the data presented in this study, we hypothesize that there are two populations of Sin1 in the cell. The Sin1 molecules of one population (in the following referred to as Sin1_cross_) become covalently cross-linked via their luminal regions to organic components in the SDV lumen (e.g., through isopeptide bonds, glycosidic bonds, or phosphodiester bonds). This event is part of the self-assembly process of organic components in the SDV lumen that results in a silica-forming insoluble organic matrix. Being part of the silica-forming organic matrix, the luminal regions of the Sin1_cross_ population become encapsulated by silica during silica biogenesis in the SDV lumen. In contrast, the molecules of the other Sin1 subpopulation only loosely interact with the components of the silica-forming organic matrix and do not become encapsulated by silica. We assume that, during exocytosis, both the cytosolic domain and the transmembrane helix of the Sin1_cross_ molecules are cleaved off and are then rapidly proteolytically degraded, whereas the luminal region becomes an integral component of the extracellular biosilica. The other population of Sin1 molecules retain their membrane anchors, and can diffuse throughout the plasma membrane–SDV membrane continuum during exocytosis.

The fate of the SDV membrane after biosilica exocytosis has thus far remained a conundrum. Four scenarios have been suggested [7], namely (1) fusion of the proximal SDV membrane with the plasma membrane, and secretion of vesicles consisting of the distal SDV membrane and the plasma membrane in the region of the cleavage furrow; (2) the same scenario as in (1), but instead of secretion of membrane vesicles the membrane material becomes an organic coat around the distal surface of the biosilica; (3) the entire SDV membrane and the plasma membrane of the cleavage furrow region become an organic coat around the entire biosilica; (4) the proximal SDV membrane fuses with the plasma membrane, whereas the distal SDV membrane is retrieved into the cytoplasm as endocytic vesicles. Previously, no experimental evidence has been provided for any of these scenarios. Our data from quantitative analysis of Sin1-GFP^C^ localization (Fig. 5a) rule out scenario (1) as we did not observe an increase of GFP fluorescence outside the cells following biosilica exocytosis of valves and girdle bands. Instead, a substantial fraction of the SDV membrane appears to become integrated into the plasma membrane. This can be concluded from the observation that the increase of GFP fluorescence throughout the plasma membrane region during valve exocytosis (from 0.20 relative fluorescence units (RFU) to 0.42 RFU) accounted for a considerable fraction (~ 25%) of the Sin1-GFP^C^ molecules that had accumulated in the SDV membrane during valve biogenesis (increase of GFP fluorescence from 0.27 RFU to 1.13 RFU). The observation is consistent with scenarios (2) and (4) and rules out scenario (3), because the latter would require all GFP fluorescence in the mid-cell region to remain associated with the valve biosilica during and after exocytosis, which is not the case (Fig. 5a, red trace at t > 0 min). Only scenario (4) is fully consistent with all our data from quantitative analysis of Sin1-GFP^C^ fluorescence as during valve exocytosis, GFP fluorescence quickly increased in the cytoplasm (from 0.16 to 0.28 RFU) suggesting the retrieval of SDV membrane by endocytic vesicles. Nevertheless, we would like to point out that our conclusions on the fate of the SDV membrane are based on the observation of just a single component of the SDV membrane, Sin1. At this point, it is unclear whether or not Sin1 is representative of all components of the SDV membrane. Therefore, additional SDV membrane proteins and lipids need to be identified to be able to accurately investigate the fate of the SDV membrane during biosilica exocytosis.

It is reasonable to assume that the function of Sin1 inside the SDVs will largely depend on the properties of the luminal domain, which represents most of the Sin1 polypeptide. Based on the results from our studies on the recombinant luminal domain, rSin1^lum^, it is likely that the Sin1 molecules will form clusters on the membrane surface driven by the low pH-induced self-aggregation of the luminal domain (Fig. 6). The shape of the clusters may be isotropic (i.e., circular patches on the membrane surface), which is the natural tendency of the luminal domain (Fig. 7). Alternatively, anisotropically shaped, homomeric or heteromeric clusters may be formed due to the presence of the transmembrane anchor in Sin1, and through interactions with other components in the SDV. It may also be possible that Sin1 covers the entire luminal surface of the SDV membrane, thereby physically separating the luminal space from the membrane. Distinguishing between these three possibilities should be feasible through future experiments localizing Sin1 by super-resolution fluorescence microscopy in vivo. We hypothesize that the shapes and arrangement of Sin1 containing clusters on the luminal surface of the SDV membrane is an important determinant for silica morphogenesis. The effect of Sin1 on silica morphogenesis may be exerted via two mechanisms. Firstly, Sin1 clusters may be involved in orchestrating the assembly of the nanopatterned insoluble organic matrix in the SDV lumen. Secondly, through interaction with LCPA molecules, which also bind to silaffins and silacidins [14, 15], Sin1 molecules could mediate the non-covalent binding of aggregates of the soluble components to the surface of the insoluble organic matrix. Aggregates of silaffin-LCPA [14] and silacidin-LCPA [15] have silica forming activities, and thus the distribution pattern of Sin1 within the insoluble organic matrix would define sites of enhanced silica forming activity. The model could be tested by determining both the distribution pattern of Sin1 in the isolated organic matrix and the binding sites for LCPA molecules. This could be achieved by immunogold electron microscopy or super-resolution fluorescence microscopy using suitable tags for Sin1 and LCPA.

## Conclusions

The present work has provided unprecedented insights into the intracellular locations of a biomineralization protein, Sin1, during silica biogenesis. Sin1 is the first identified SDV membrane protein, and its interaction with LCPA suggests a mechanism by which the SDV membrane could influence silica morphogenesis in the SDV lumen. It is to be expected that diatom SDVs contain additional membrane proteins besides silicanins. In 2015, at the meeting “Molecular Life of Diatoms” (in Seattle, WA, USA), a family of putative SDV membrane proteins from *T. pseudonana* were reported by the group of Mark Hildebrand (Scripps Institution of Oceanography, UCSD, USA). These proteins also contain predicted transmembrane domains, but show no sequence similarity to Sin1 (Mark Hildebrand, personal communication). In future work, appropriately tagged Sin1 could be used as a bait for identification of Sin1 interacting proteins, and possibly also enable a next big step in silica biomineralization research, namely the isolation of SDVs. Such achievement would allow for in depth characterization of the biomolecular composition of SDVs and studies of their interactions with the cytoskeleton and other cellular components.

## Methods

### Chemicals, enzymes, and antibodies

Oligonucleotides were purchased from Eurofins Genomics, isopropylthiogalactoside from Carl Roth, ampicillin from Merck, nourseothricin from Jena Bioscience, tetramethyl orthosilicate (TMOS) from Sigma-Aldrich, and enzymes used for molecular genetics were from obtained Thermo Scientific. Ammonium molybdate tetrahydrate, NH_4_F, 37% HCl, ethylenediamine tetraaecetic acid (EDTA), and sodium dodecylsulfate (SDS) were purchased from Merck. Anti-rSin1^lum^ antibodies were produced by Pineda Antibody-Service through immunization of rabbits with SDS-PAGE purified rSin1^lum^. A polyclonal anti-GFP antibody (from rabbits) against full-length GFP was obtained from Clontech. Anti-PsbD and anti-AtpB antibodies (both produced in rabbits) were purchased from Agrisera. Anti-rabbit IgG from goat (whole molecule, peroxidase-conjugate) was purchased from Sigma Aldrich. An AlexaFluor647-conjugated anti-rabbit IgG antibody from goat was obtained from Thermo Fisher Scientific. MilliQ-purified H_2_O (resistivity: 18.2 MΩ∙cm) was used throughout this study.

### Culture conditions

*Thalassiosira pseudonana* (Hustedt) Hasle et Heimdal clone CCMP1335 was grown in an enriched artificial seawater medium (EASW) according to the North East Pacific Culture Collection protocol (Canadian Center for the Culture of Microorganisms ESAW Recipe) at 18 °C under constant light at 5000–10,000 lux.

### Cloning, expression and purification of rSin1^lum^ and rSin1^-SP^

The DNA sequence of Sin1 is present in the Uniprot database under ID B8CBQ8. The DNA sequences encoding for amino acids 25–383 of Sin1 (rSin1^lum^) and amino acids 25–426 of Sin1 (rSin1^-SP^) were amplified from *T. pseudonana* cDNA, including a hexahistidine coding sequence at the 3’-ends. The resulting PCR products were incorporated into the expression vector pJ404 (DNA2.0) as described in Additional file 1: Supporting Materials and Methods. Expression and purification of the recombinant proteins from *E.coli* DH5α is described in detail in Additional file 1: Supporting Materials and Methods.

### Isolation of LCPA

*T. pseudonana* LCPA were isolated by modification of a previously published method [14]. *T. pseudonana* biosilica (~ 1.2 g) was isolated by SDS/EDTA extraction and incubated with 96 mL 10 M NH_4_F (adjusted to pH 4.5 with 44 mL 6 M HCl) for 1 h at room temperature to dissolve the silica. After centrifugation (30 min, 3200 *g*), the supernatant was filtered through a polyethersulfone syringe filter (pore size 0.2 μm; Carl Roth), and desalted against 200 mM ammonium acetate using a HiPrep 26/10 desalting column (column volume 53 mL, GE Healthcare) injecting 10 mL of NH_4_F-soluble material per run (flow rate: 9 mL min^–1^). Fractions eluting between 1.3 and 3.6 min after sample injection were combined and freeze-dried. The residue was dissolved in 5.5 mL H_2_O, centrifuged (5 min, 20,000 *g*), and subjected to gel filtration chromatography on a Superose 12 10/300 GL column (GE Healthcare) equilibrated with 200 mM ammonium acetate (flow rate: 0.5 mL min^–1^). Fractions eluting between 16.5 min and 20 min contained complexes of silaffins and LCPA; they were combined and freeze-dried. The residue was dissolved in 200 mM ammonium acetate supplemented with 2 M NaCl to disrupt the electrostatic interactions between silaffins and LCPA. After centrifugation (5 min, 20,000 *g*), the supernatant was subjected to gel filtration chromatography on a Superdex Peptide 10/300 GL column (GE Healthcare) equilibrated with 200 mM ammonium acetate (flow rate: 0.25 mL min^–1^). Fractions eluting between 55 and 61.5 min were combined, freeze-dried, and dissolved in H_2_O. Analysis by SDS-PAGE with Coomassie and “Stains All” staining confirmed the purity of LCPA. The concentration of LCPA was determined using the 660 nm Protein Assay (Pierce) with the synthetic oligopropyleneimine dendrimer DAB-Am-16 (Sigma-Aldrich) as a standard.

### Structural and functional characterization of rSin1^lum^

#### DLS

All protein-free solutions were filtered through a polyethersulfone membrane (pore size 0.2 μm; Carl Roth), and stock solutions of rSin1^lum^ were centrifuged (5 min, 20,000 *g*). The centrifuged rSin1^lum^ stock solution was adjusted to a protein concentration of 1 mg mL^–1^ by dilution in 10 mM sodium phosphate-citrate at pH 7.7. The solution was incubated at room temperature for 1 hour prior to measurements with a Zetasizer Nano-ZS instrument (Malvern Instruments, UK). The pH of the rSin1^lum^ solution was acidified by dropwise addition of 50 mM citric acid, and finally neutralized by dropwise addition of 100 mM NaOH (note, after all pH adjustments the increase of sample volume was < 10%). After each pH change, the protein solution was incubated for 1 hour at room temperature, and then measured in a quartz cuvette with 10 mm path length at 25 °C using the 173° Backscatter option of the instrument with automatic determination of the measurement duration. Data processing was performed using the protein analysis model of the Zetasizer software. Data from five measurement runs were averaged.

#### Cryo-transmission electron microscopy

All protein-free solutions were filtered through a polyethersulfone membrane (0.2 μm pore size) to remove undissolved particles. The stock solution of rSin1^lum^ (4.8 mg mL^–1^) in 10 mM Na-phosphate pH 7.7 was centrifuged (5 min, 20,000 *g*), diluted to 1.0 mg mL^–1^ and adjusted to the desired pH using 50 mM citric acid. Sample vitrification for Cryo-TEM was carried out using an automated vitrification robot (Vitrobot™ Mark III, FEI). Sample supports (type R2/2 Quantifoil), were purchased from Quantifoil Micro Tools GmbH and contained a carbon support film on a copper grid. Prior to use, the TEM grids were glow-discharged in a Cressington 208 carbon coater to render them hydrophilic. Cryo-samples were prepared from a 3 μL droplet of sample solution placed on the grid inside the Vitrobot™ chamber at 100% relative humidity and temperature of 20 °C, after which it was blotted to remove excess solution and subsequently plunged into liquid ethane for vitrification. Imaging was performed using a FEI CryoTitan operating at 300 kV and equipped with a field emission gun using low dose procedures [38].

#### Circular dichroism spectroscopy

A stock solution of rSin1^lum^ was centrifuged (5 min, 20,000 *g*) and diluted to 4 μM using filtered (0.2 μm pore size) 10 mM sodium phosphate pH 7.7. Spectra were acquired in a quartz cuvette with a thickness of 1 mm (110-1-P-40; Hellma Analytics) using a Chirascan-Plus CD spectrometer (Applied Photophysics) set to 25 °C or 93 °C. Measurement parameters were five accumulations per measurement, 260–190 nm wavelength range, 1 nm wavelength step size, 1 nm bandwidth, 0.5 s time per point. Raw spectra of rSin1^lum^ were corrected by subtracting a spectrum of the buffer solution. Data were then transformed into the mean residue ellipticity (MRE, physical dimension: deg cm^2^ dmol^–1^) by applying the equation MRE = θ × 100/(c × d × AA), where θ is the measured ellipticity (in degrees), c is the protein concentration (in dmol mL^–1^), d is the path length of the cuvette (in cm), and AA is the number of amino acid residues in rSin1^lum^. The transformed data (wavelength range 240–190 nm) were then analyzed for secondary structure content using the DiChroWeb server [39, 40] and the CDSSTR method (reference data set 7 from taken from reference [41]).

#### Ellman’s assay for thiol groups

The assay was performed following a protocol provided by the manufacturer of Ellman’s reagent (5,5′-dithio-bis-(2-nitrobenzoic acid, Thermo Scientific). Different concentrations of rSin1^lum^ in 10 mM sodium phosphate buffer pH 7.7 were mixed with 180 μM Ellman’s reagent followed by a 15 min incubation period and photometric detection at 412 nm using a plate reader (Biotek). Cysteine hydrochloride was used to generate a standard curve, which was then used to calculate the concentration of thiol groups in a given concentration of rSin1^lum^.

#### Silica formation assay

Stock solutions of rSin1^lum^ and LCPA were diluted to a final concentration of 25 μM in 50 mM sodium acetate at pH 5.5. Where required, mixtures were supplemented with 30 mM sodium phosphate-citrate at pH 5.5. Silicic acid was freshly prepared by hydrolysis of TMOS (1 M TMOS in 1 mM HCl, 15 min at room temperature under constant mixing) and added to the protein solutions at a final concentration of 100 mM. After 10 min incubation, the solutions were centrifuged (5 min, 16,000 *g*) and the pellets were washed three times with H_2_O followed by centrifugation (5 min, 16,000 *g*). The final pellet was dissolved in 2 M NaOH (95 °C, 1 hour), and the silica concentration determined using the silicomolybdate assay [42].

#### Expression of GFP-tagged Sin1 in *T. pseudonana*

The start and stop codons of Sin1 were confirmed by 5’- and 3’-RACE PCR (Additional file 1: Figure S10, experimental details in Supporting Materials and Methods). Construction of the fusion genes encoding Sin1-GFP^C^ and Sin1-GFP^N^ and their expression in *T. pseudonana* is described in Additional file 1: Supporting Materials and Methods.

#### Expression of double-tagged Sin1 in *T. pseudonana*

Construction of the Sin1-mT2^N^-Venus^C^ fusion gene (mTurquoise2 located between the RXL and NQ-rich regions of the luminal domain and Venus located at the C-terminus of the cytosolic domain of Sin1) is described in Additional file 1: Supporting Materials and Methods.

#### Fluorescence microscopy of biosilica and organic matrices

Biosilica and insoluble organic matrices were isolated from *T. pseudonana* as described previously [17], and analyzed for GFP by epifluorescence microscopy using a 63× oil objective on a Zeiss Axiovert 200 inverted microscope equipped with a Piston filter (Chroma; excitation 450–490 nm, emission 500–530 nm).

#### Confocal fluorescence microscopy of live cells

For imaging, 10 μL of a cell suspension was transferred onto a 22 mm × 50 mm coverslip and covered with a rectangular slice (~ 0.5 cm^2^) made of 1% (w/v) agarose in EASW medium. Images were acquired using a Zeiss LSM780 inverted confocal microscope equipped with a Zeiss Plan-Apochromat 63× (1.4) Oil DIC M27 objective. GFP fluorescence and chlorophyll autofluorescence were detected in one-track mode using an Argon laser line (power set to 2%), a MBS 488 beam splitter and a 32-channel GaAsP spectral detector. Two channels were acquired to separately monitor the GFP fluorescence (emission at 491–535 nm) and chloroplast fluorescence (emission at 654–693 nm). Images of double-tagged Sin1 (Sin1-mT2^N^-Venus^C^) were acquired using the 440 nm (for mTurquoise2) and 514 nm (for Venus and chloroplasts) laser lines set to laser intensities of 0.2% and 1%, respectively. Three channels were acquired to separately monitor the fluorescence of mTurquoise2 (emission at 455–500 nm), Venus (emission at 517–553 nm) and chloroplast fluorescence (emission at 657–688 nm). All images were analyzed using the ZEN2012 software (Zeiss).

For time-lapse imaging, equal volumes of a cell suspension and an approximately 30 °C solution of 1% (w/v) low-melting agarose (Fisher Scientific, USA) in EASW medium were gently mixed and transferred into a 35 mm diameter petri dish with a glass bottom (1.5H, 170 ± 5 μm; Ibidi, Germany). To allow for gelation, the petri dish was incubated at 18 °C for 10 min. When labeling with PDMPO (Biomol, Germany) was required, 190 μL of 10 μM PDMPO in EASW medium was mixed with 750 μL cell suspension and the resulting suspension was mixed with 940 μL of agarose as described above, yielding a gel with a final PDMPO concentration of 1 μM. After overlaying 2 mL of EASW medium on top of the agarose gel, the petri dish was sealed with Parafilm and incubated for approximately 2 hours at room temperature in the dark before mounting onto a microscope stage tempered at 17 °C (Thermal Insert and Liquid Cooling System from Warner Instruments, USA). Imaging was performed with an inverted IX81 microscope (Olympus, Japan) equipped with an UApochromat 60× 1.15 W air objective, ZDC hardware autofocus, NanoScanZ and ProScanIII xy scanning stages (Prior Scientific), and diode 405 nm, DPSS 488 nm, and 561 nm lasers (Coherent). GFP (ex. 488 nm, em. 525/30 nm bandpass), PDMPO (ex. 405 nm, em. 525/30 nm bandpass), and chlorophyll autofluorescence (excitation 488 nm, emission 568 nm longpass) were detected using a spinning disc Yokogawa CSU-X1 dichroic beam splitter (5000 rpm; Triple band T-405/488/561) and an iXon EM+ DU-897 BV back illuminated EMCCD camera (Andor, Oxford Instruments). For each experiment, a total of 15 agarose-embedded cells were selected for imaging. Each cell was exposed to LED brightfield light (50 ms) and then z-scanned (± 2 μm around the center of the cell with 0.5 μm steps yielding 9 z-scans per cell per time point) with the following lasers in the order given: 488 nm (chlorophyll; laser power: ~23 μW; t = 50 ms), 488 nm (GFP; laser power: ~115 μW; t = 600 ms), 405 nm (PDMPO; laser power: ~37 μW; t = 150 ms). This procedure was subsequently repeated cell by cell. Cells were imaged in the same order in 3.5 min intervals for a total of 100 times. Labeling with 1 μM Hoechst 34580 dye (Thermo Scientific, USA) was performed as described for PDMPO above, and imaged by exciting at 405 nm (laser power: ~56µW, t = 150 ms) and detecting emission at 445 nm.

#### Quantitative analysis of fluorescence intensities from time lapse imaging

The raw movie files were analyzed using Fiji (ImageJ, National Institute of Health) and Matlab R2016a (Mathworks). To examine valve biogenesis, live-cell imaging data of four cells were analyzed as follows. The raw movies were rotated and cropped to align the cells vertically. The intensities of all nine z-planes recorded were summarized for each frame yielding Fig. 5. The background was corrected by setting the global minimum fluorescence intensity outside the cell to zero. The movie was further processed in Matlab correcting for drift via a cross-correlation image registration [43]. The movie was then split into rectangular subregions as indicated in the corresponding figures. The region size was linearly interpolated between key frames to account for cell growth during the recording time. The fluorescence intensity was summarized for each region and frame, and plotted over time. The total fluorescence intensity prior to cytokinesis was normalized to 1.0 to allow for comparison of different cells. To build an average intensity plot of all cells, the peak intensity for PDMPO fluorescence was used for post-synchronization. Time-lapse data from four cells were used to generate an average fluorescence intensity plot.

#### Preparation of membranes from *T. pseudonana*

Total membranes were prepared according to a published protocol [21].

#### Carbonate extraction of total membranes from *T. pseudonana*

Equal aliquots of freshly prepared membranes were resuspended in carbonate buffer (0.1 M NaHCO_3_, 1 mM EDTA, pH 11.5) or lysis buffer (50 mM Hepes, 150 mM NaCl, 250 mM sucrose, pH 7.5). All buffers contained one tablet of EDTA-free protease inhibitor (Pierce) per 10 mL of buffer. The membrane suspensions were incubated on ice for 45 min, followed by centrifugation at 100,000 *g* for 60 min. The supernatants were collected and the membrane pellets were resuspended in lysis buffer. Equal aliquots of supernatant and membrane pellet were analyzed by Western Blot using antibodies directed against rSin1^lum^, PsbD, and AtpB (see below).

#### HF treatment of membranes from *T. pseudonana*

Membranes were freeze-dried, mixed with approximately 500 μL anhydrous HF (GHC Gerling), and incubated on ice for 60 min. The HF was removed by evaporation using a gentle stream of nitrogen followed by drying in a Speedvac. The residue was resuspended in 150 μL lysis buffer (50 mM Hepes, 150 mM NaCl, 250 mM sucrose, pH 7.5) supplemented with 1% Igepal, and immediately neutralized using NH_4_OH. The suspension was mixed with sample loading solution and analyzed by Western blot using the anti-Sin1 antibody as described below.

#### Western blot analysis

For SDS-PAGE aliquots of the solubilized membranes were mixed with sample loading buffer, incubated for 10 min at 95 °C or 60 °C (membrane containing samples), and centrifuged (5 min, 20,000 *g*) prior to loading on NuPAGE 4–12% Bis-Tris SDS-PAGs (Thermo Scientific). For Sin1, detection gels were wet-blotted onto Protran 0.45 μm nitrocellulose membranes (GE Healthcare) and for PsbD and AtpB detection onto 0.45 μm PVDF membranes (Immobilon-P, Millipore) using Towbin buffer (25 mM Tris, 192 mM glycine) supplemented with 20% (v/v) methanol. The blots were blocked with 2% (w/v) albumin fraction V (Merck Millipore) dissolved in TBST (50 mM Tris, 150 mM NaCl, 0.05% (v/v) Tween-20 (Biorad), pH 7.5). After 60 min incubation at room temperature, the blots were washed three times for 10 min with 20 mL TBST. The blots were then incubated with the desired antibodies diluted in TBST (1:10,000 anti-rSin1^lum^ antiserum, 1:2500 anti-PsbD and anti-AtpB antibodies). After 60 min incubation at room temperature, the blots were washed three times for 10 min with 20 mL TBST and subsequently incubated with anti-rabbit IgG (Sigma) dissolved in TBST at a dilution of 1:10,000. After 60 min incubation at room temperature, the blots were washed twice in 20 mL TBST and twice in 20 mL TBS (i.e., TBST without Tween-20). Excess buffer was removed from the blots using paper towels before incubating with 2 mL SuperSignal West Pico chemiluminescent substrate (Thermo Scientific) for 5 min at room temperature. Chemiluminescence was detected using the ChemiDoc MP imaging system (Biorad).

#### Antibody accessibility experiments

Quantification of the accessibility of Sin1 in biosilica and organic matrices from Sin1-GFP^N^-expressing transformants was performed with a 1:1000 dilution of the anti-Sin1 antiserum using a previously published method [17]. The method is described in detail in Additional file 1: Supporting Materials and Methods.

#### Immunodetection of GFP in biosilica and insoluble organic matrices

Immunolabeling of biosilica and organic matrices from wild-type cells and transformants expressing Sin1-GFP^C^ or Sin1-GFP^N^ was carried out using an anti-GFP antibody as described in Additional file 1: Supporting Materials and Methods.

#### Bioinformatics analysis

The genomes of the diatoms *C. cryptica* [44], *T. oceanica* [45], and *F. cylindrus* [46] are publically available from the UCSC genome browser, the NCBI database, and the JGI database, respectively. Transcriptome databases for other diatom and non-diatom organisms were downloaded from the Marine Microbial Eukaryote Transcriptome Sequencing Project [18] (MMETSP; current number of entries in the re-assembled database used: 659; download link: https://monsterbashseq.wordpress.com/2016/09/13/mmetsp-re-assemblies/). The Sin1 (*T. pseudonana*) protein sequence was used to perform a tBlastn search against the respective databases (Matrix: Blosum62, Gap Penalties: Existence: 11, Extension: 1, Neighboring words threshold: 13, Window for multiple hits: 40). BLAST hits with an E-value of lower than approximately 1 × 10^–50^ were considered Sin1 homologous proteins, accounting for 70 unique diatom species and two non-diatom species out of the 659 entries in the MMETSP. Protein sequences of Sin1 homologues were extracted from the translated transcriptome databases for the two non-diatom species and three species each from centric diatoms and pennate diatoms that showed the lowest E-values. These protein sequences were then used to calculate sequence identities to Sin1 and to prepare the accompanying sequence alignment in Additional file 1: Figure S1. Blast searches were also performed using the genome of *Amphimedon queenslandica* [20], which is available at the NCBI database, and the transcriptomes of *Haliclona amboinensis* and *Ephydatia muelleri*, which are both available at http://www.compagen.org.

## Additional files

Additional file 1: Figure S1. Sequence conservation of silicanins in diatoms and other protists. **Figure S2**. DNA and amino acid sequences of rSin1^-SP^ and rSin1^lum^, **Figure S3**. Membrane association of Sin1. **Figure S4**. Accessibility of Sin1 in biosilica and organic matrices. **Figure S5**. Localization of a double-tagged Sin1 fusion protein (Sin1-mT2^N^-Venus^C^) in *T. pseudonana*. **Figure S6**. Anti-GFP immunolabeling of biosilica and organic matrices isolated from *T. pseudonana* transformant cells expressing Sin1-GFP^C^ or Sin1-GFP^N^. **Figure S7**. Time-lapse imaging of a live cell around the time of nuclear division. **Figure S8**. Time-dependent quantitative analysis of region-specific GFP fluorescence during girdle band formation in a Sin1-GFP^C^ expressing cell. **Figure S9**. Biochemical analysis of rSin1^lum^. **Figure S10**. DNA sequences obtained from 5’- and 3’-RACE PCR analysis of Sin1 mRNA. **Table S1**. Sin1 homologues identified through tBLASTn searches. **Table S2**. Sequence identities between *T. pseudonana* (Tps) Sin1 and Sin2, and between homologues from other diatoms and non-diatom organisms. **Table S3**. Quantification of the extractability of Sin1, PsbD, and AtpB from *T. pseudonana* membranes. **Table S4**. Quantification of the accessibility of Sin1 in biosilica. **Table S5**. Secondary structure analysis of rSin1^lum^. **Table S6**. Determination of free sulfhydryl groups in rSin1^lum^. (PDF 1979 kb)

Additional file 2: Movie S1. Time-lapse imaging of a Sin1-GFP^C^ expressing live cell around the time of valve biogenesis. (AVI 21692 kb)

Additional file 3: Movie S2. Time-lapse imaging of a live-cell around the time of nuclear division. (AVI 21692 kb)

Additional file 4: Movie S3. Time-lapse imaging of a live cell around the time of girdle band biogenesis. (AVI 7201 kb)

## Abbreviations

BLAST: basic local alignment search tool
DLS: dynamic light scattering
EASW: enriched artificial sea water
EDTA: ethylenediamine tetraacetic acid
GFP: enhanced green fluorescent protein
LCPA: long-chain polyamines
MMETSP: Marine Microbial Eukaryote Transcriptome Sequencing Project
MRE: mean residue ellipticity
mT2: mTurquoise2
PAGE: polyacrylamide gel electrophoresis
PDMPO: 2-(4-pyridyl)-5-((4-(2-dimethylaminoethylaminocarbamoyl)methoxy)phenyl)oxazole
SDS: Sodium dodecylsulfate
SDV: silica deposition vesicle
Sin1: Silicanin-1
TMOS: tetramethoxysilane
