# Supplementary material for: Silicanin-1 is a conserved diatom membrane protein involved in silica biomineralization
Source: BMC Biol. 2017 Jul 24;15:65. doi: 10.1186/s12915-017-0400-8 (PMC5525289; doi:10.1186/s12915-017-0400-8)
Supplement: Supplementary file 1 — Sequence conservation of silicanins in diatoms and other protists. Figure S2. DNA and amino acid sequences of rSin1-SP and rSin1lum, Figure S3. Membrane association of Sin1. Figure S4. Accessibility of Sin1 in biosilica and organic matrices. Figure S5. Localization of a double-tagged Sin1 fusion protein (Sin1-mT2N-VenusC) in T. pseudonana. Figure S6. Anti-GFP immunolabeling of biosilica and organic matrices isolated from T. pseudonana transformant cells expressing Sin1-GFPC or Sin1-GFPN. Figure S7. Time-lapse imaging of a live cell around the time of nuclear division. Figure S8. Time-dependent quantitative analysis of region-specific GFP fluorescence during girdle band formation in a Sin1-GFPC expressing cell. Figure S9. Biochemical analysis of rSin1lum. Figure S10. DNA sequences obtained from 5’- and 3’-RACE PCR analysis of Sin1 mRNA. Table S1. Sin1 homologues identified through tBLASTn searches. Table S2. Sequence identities between T. pseudonana (Tps) Sin1 and Sin2, and between homologues from other diatoms and non-diatom organisms. Table S3. Quantification of the extractability of Sin1, PsbD, and AtpB from T. pseudonana membranes. Table S4. Quantification of the accessibility of Sin1 in biosilica. Table S5. Secondary structure analysis of rSin1lum. Table S6. Determination of free sulfhydryl groups in rSin1lum. (PDF 1979 kb) [file 12915_2017_400_MOESM1_ESM.pdf]

Supporting Information

Title: Silicanin-1 is a conserved diatom membrane protein involved in silica biomineralization

Authors: Alexander Kotzsch, Philip Gröger, Damian Pawolski, Paul H. H. Bomans, Nico A. J. M. Sommerdijk, Michael Schlierf, Nils Kröger

Supporting Material and Methods

Cloning, expression and purification of rSin1^{lum}. The DNA sequence encoding for amino acids 25-383 of Sin1 (Uniprot ID B8CBQ8) was amplified from *T. pseudonana* cDNA using the sense primer 5'-TTACCATATGAACACCAAAAAGGCCGC (*Nde*I site underlined) and the antisense primer 5'-ATAGGGATCC**TTA***GTGGTGGT GGTGATGGTG*ACCAGTAGTGGCAACTCC (*BamH*I site underlined, His_6 tag in italics, stop codon in bold; Fig. S2b). The PCR product was incorporated into expression vector pJ404 (T5 promoter, ampicillin resistance, IPTG inducible; DNA2.0) via the *Nde*I and *BamH*I restriction sites yielding the plasmid pJ404-rSin1^{lum}, which was introduced into chemically competent *E. coli* DH5α cells. For protein expression, 300 mL of LB medium containing 100 µg·mL^{-1} ampicillin were inoculated with a DH5α transformant clone carrying the pJ404-rSin1^{lum} plasmid, and grown in a shaker-incubator at 37°C and 180 rpm overnight. The following day, 6 L of LB medium containing 100 µg·mL^{-1} ampicillin was inoculated with 240 mL of the overnight culture and grown at 37°C and 180 rpm. At an OD (at 600 nm) of ~0.6 the culture was supplemented with 1 mM IPTG and further incubated for 3 hours. Cells were harvested by centrifugation (6,000 xg, 30 min, 4°C). The cell pellet was washed with 120 mL 1% NaCl, weighed, and stored at -80°C.

Cell lysis was achieved by resuspending frozen cells (~ 28 g) in 200 mL of lysis buffer (50 mM Hepes-NaOH pH 7.5, 0.5 M NaCl) supplemented with 1 mM PMSF and 3 tablets EDTA-free protease inhibitor (Pierce), and processed using an emulsifier (EmulsiFlex-C3, Avestin) tempered to 4 °C. The ice-cold cell suspension was passed through the emulsifier for a total of four times applying a pressure of 15,000-20,000 psi. After the last round of lysis, the emulsifier was washed with 20 mL lysis buffer. The lysate and wash fraction were centrifuged (12,000 xg, 20 min, 4°C). As rSin1^{lum} was present in inclusion bodies, the following conditions were used to solubilize and renature rSin1^{lum}. The inclusion bodies were washed three times with 4.5 volumes of lysis buffer containing 2 M urea and centrifuged (12,000 xg, 20 min, 16°C). The resulting pellet was carefully resuspended in 6 volumes lysis buffer containing 8 M urea and stirred at room temperature for 16 hours. The solution was then centrifuged (12,000 xg, 20 min, 16°C) and the supernatant was subjected to immobilized metal affinity chromatography using 5 mL HisPur Ni-NTA resin (Thermo Scientific). The resin was equilibrated in lysis buffer, subsequently mixed with the rSin1^{lum} containing solution, and incubated at room temperature for 2 hours under constant mixing. The suspension was poured into a glass column that was equipped with a frit (Sigma Aldrich), the resin was washed twice with four column volumes of washing buffer 1 (50 mM Hepes-NaOH pH 7.5, 0.5 M NaCl, 8 M urea), and three times with two column volumes washing buffer 2 (50 mM Hepes-NaOH pH 7.5, 0.5 M NaCl, 2 M urea, 25 mM Imidazole). Elution of rSin1^{lum} was achieved by rinsing the columns with 6 column volumes elution buffer (50 mM Hepes-NaOH pH 7.5, 0.5 M NaCl, 2 M urea, 250 mM Imidazole). To allow for refolding of rSin1^{lum} and formation of the native disulfide bonds the combined elution fractions were dialyzed twice against 2 L renaturation buffer 1

(20 mM Tris-HCl, 300 mM NaCl, 5% glycerol, 2.5 mM reduced glutathione, 0.25 mM oxidized glutathione, pH 8.5) and twice against 2 L renaturation buffer 2 (20 mM Tris-HCl, 300 mM NaCl, 5% glycerol, pH 8.5) at 4°C for 24 hours in each dialysis buffer. Finally, the protein solution was dialyzed against 2 L MQ buffer 1 (20 mM Tris-HCl pH 8.5) at 4 °C for 24 hours.

The dialysate was subjected to anion-exchange chromatography using a MonoQ 5/50 column (GE Healthcare) equilibrated in MQ buffer 1. The dialysate was loaded onto the column at a flow rate of 0.5 mL min^{-1}, the column was washed with MQ buffer 1 for 30 min at the same flow rate, and then subjected to a linear gradient (48 min, 0.5 mL min^{-1} flow rate) from 100% MQ buffer 1 to 50% MQ buffer 2 (20 mM Tris-HCl pH 8.5, 2 M NaCl) followed by a 7 min linear gradient to 100% MQ buffer 2. Elution of rSin1^{lum} occurred between 20-25% MQ buffer 2. Fractions were analyzed by SDS-PAGE and Coomassie Blue staining, and those containing rSin1^{lum} were combined.

As a final purification step the rSin1^{lum} containing Mono Q fractions were pooled and loaded onto a Superdex 75 10/300 (GE Healthcare) size-exclusion column and chromatographed in SEC buffer (20 mM Tris-HCl pH 8.5, 0.3 M NaCl) at a flow rate of 0.5 mL min^{-1}. Superdex fractions containing pure rSin1^{lum} (analyzed by SDS-PAGE) were combined and dialyzed against storage buffer (10 mM sodium phosphate pH 7.7) using regenerated cellulose dialysis tubing (10 kDa cutoff; SpectraPor, Spectrum Labs). Aliquots were flash-frozen and stored at -80 °C (note: for cryo-TEM studies rSin1^{lum} was used directly after purification without freezing). The concentration of rSin1^{lum} was determined by measuring the absorbance at 280 nm using the calculated molar extinction coefficient of 42,385 M^{-1} cm^{-1}.

Cloning and expression of rSin1^{-SP}. The DNA sequence encoding for amino acids 25-426 of Sin1 (Uniprot ID B8CBQ8) was amplified from *T. pseudonana* cDNA using the sense primer 5'-TTACCATATGAACACCAAAAAGGCCGC (*Nde*I site underlined) and the antisense primer 5'-ATAAGGATCC**TTA***GTGATGATGGTGATGGTG* AGCCATGGCACCACCCTGTC (*BamH*I site underlined, His_6 tag in italics, stop codon in bold; Fig. S2a). The PCR product was incorporated into expression vector pJ404 (T5 promoter, ampicillin resistance, IPTG inducible; DNA2.0) via the *Nde*I and *BamH*I restriction sites yielding the plasmid pJ404-rSin1^{-SP}, which was introduced into chemically competent *E. coli* DH5α cells. For protein expression, 5 mL of LB medium containing 100 µg·mL^{-1} ampicillin were inoculated with a DH5α transformant clone carrying the pJ404-rSin1^{-SP} plasmid, and grown in a shaker-incubator at 37°C and 180 rpm overnight. The following day 25 ml of LB medium containing 100 µg·mL^{-1} ampicillin was inoculated with 0.25 mL of the overnight culture and grown at 37°C and 180 rpm. At an OD (at 600 nm) of ~0.9 the culture was supplemented with 1 mM IPTG and further incubated for 3 hours. Cells were harvested by centrifugation (6,000 xg, 10 min, 4°C). The cell pellet was washed with 20 mL 1% NaCl and stored in aliquots at -80°C. For SDS-PAGE one aliquot of cells was resuspended in 100 µl sample loading buffer (SDS-PAGE) and incubated at 95 °C for 10 min. The resulting mixture was diluted 50-

fold with sample loading buffer and 15 µl of the diluted rSin1^{-SP} cell lysate were used as control in Western blots (see Material and Methods section in the main text).

Expression of GFP-tagged Sin1 in T. pseudonana. The start and stop codons of Sin1 were confirmed by 5'- and 3'-RACE PCR (Fig. S10). Total mRNA was isolated from *T. pseudonana* using oligo-dT-functionalized magnetic beads, and cDNA synthesis was performed as described previously (1). For amplification of the 3'-end, nested PCRs were performed using the gene-specific sense primer 5'-TCTTGGAAGTG TTGGACTTGC and the antisense primer 5'-GGCCACGCGTCGACTAGTAC$(T)_{17}$ for the first PCR, and the gene-specific sense primer 5'-ATATGCTGCCATGTTGCACC and the antisense primer 5'-GGCCACGCGTCGACTAGTAC for the second PCR. To amplify the 5'-end, two nested PCRs were performed using sense primer 5'-GGCCACGCGTCGACTAGTACGGGIIGGGIIGGGIIG and the gene-specific antisense primer 5'-GAATTTGACAGAGTATCCGCTG for the first PCR, and sense primer 5'-GGCCACGCGTCGACTAGTAC and gene specific antisense primer 5'-ATCGTACGCATCCTCGAG for the second PCR. All PCR products were ligated to the pJet1.2 vector (Thermo Scientific) and sequenced.

For C-terminal GFP-tagging, the Sin1 terminator region (673 bp downstream of the stop codon) was amplified from genomic DNA using the sense primer 5'- ATGGCT GCGGCCGCTTAAGTCTCGTTTGAAG-3' and the antisense primer 5'- AAGAGG *GTTAAC*GTCGAGGGTGTTCTGAAAG-3' (*Not*I site underlined and *Hpa*I site in italics). The resulting PCR product was digested with *Not*I and *Hpa*I and introduced into the *Not*I and *Hpa*I sites of pTpNR-GFP_{HpaI}/fcpNat(-*Not*I) (2) generating pPnr-GFP-TSin1/fcpNAT(-*Not*I). The promoter region (1013 bp upstream of the start codon) and protein coding region of Sin1 were amplified from genomic DNA using the sense primer 5'-TCGTACTTGGGCCCATACTCCTGGTCC-3' and the antisense primer 5'-AAACG A*GGTACC*AGCCATGGCACCACCC -3' (*Apa*I site underlined, *Kpn*I site in italics) and introduced into the *Apa*I and *Kpn*I sites of pPnr-GFP-TSin1/fcpNAT(-*Not*I) generating the final expression plasmid pPSin1-Sin1-GFP^{C}-TSin1/fcpNAT(-*Not*I). In this plasmid Sin1 is expressed with a C-terminal GFP-tag (Sin1-GFP^{C}) under control of the endogenous Sin1 regulatory sequences.

For N-terminal GFP-tagging, the GFP gene was inserted directly downstream of the RRL motif of Sin1. The Sin1 coding region downstream of the RRL motif (covering amino acids 48-426, "Part2") including the terminator region were amplified from genomic DNA using the sense primer 5'- TCGCCGCGGCCGCTGCGTACGAT GGCCAAG-3' and the antisense primer 5'- AAGAGG*GTTAAC*GTCGAGGGTGTTC TGAAAG-3' (*Not*I site underlined and *Hpa*I site in italics). The resulting PCR product was digested with *Not*I and *Hpa*I and introduced into the *Not*I and *Hpa*I sites of pTpNR-GFP_{HpaI}/fcpNat(-*Not*I) (3) generating pPnr-$Sin1^{Part2}$-GFP^{C}-TSin1/fcpNAT(-*Not*I). The region of the Sin1 gene upstream of the RRL motif and including the promoter region was amplified from genomic DNA using the sense primer 5'- TCGTACTTGGGCCCAT ACTCCTGGTCC-3' and the antisense primer 5'- GCCATC*GGTACC*ATCCTCG

AGGCGACG-3' (*Apa*I site underlined, *Kpn*I site in italics) and introduced into the *Apa*I and *Kpn*I sites of pPnr-Sin1^{Part2}-GFP^{C}-TSin1/fcpNAT(-*Not*I) generating the final expression plasmid pPSin1-Sin1^{Part1}-GFP^{N}-Sin1^{Part2}-TSin1/fcpNAT. This plasmid encodes a Sin1 fusion protein with the GFP-tag immediately following the RXL domain (Sin1-GFP^{N}), which is under expression control of the endogenous Sin1 regulatory sequences. Transformation of *T. pseudonana* and selection of transformants on nourseothricin containing agar plates was performed as described previously (3).

Expression of double-tagged Sin1 in T. pseudonana. For N-terminal tagging of Sin1 with mTurquoise2 (abbreviated mT2) (4), the GFP gene in plasmid pPSin1-Sin1^{Part1}-GFP^{N}-Sin1^{Part2}-TSin1/fcpNAT (see above) was replaced by the mT2 DNA using the *Kpn*I and *Not*I restriction sites flanking the GFP gene. The mT2 DNA sequence was amplified using the sense primer 5'-AGGATGGTACCGGCGGAATGGTGAGCAAGGG CG and the antisense primer 5'-TACGCA*GCGGCCGC*TCCCTTGTACAGCTCGTCC (*Kpn*I site underlined, *Not*I site in italics) and introduced into the *Kpn*I and *Not*I sites of plasmid pPSin1-Sin1^{Part1}-GFP^{N}-Sin1^{Part2}-TSin1/fcpNAT generating pPSin1-Sin1^{Part1}-mT2^{N}-Sin1^{Part2}-TSin1/fcpNAT. Subsequently, the restriction sites for *Afl*II and *Nco*I located in the Sin1 promoter region of pPSin1-Sin1^{Part1}-mT2^{N}-Sin1^{Part2}-TSin1/fcpNAT were removed by overlap extension PCR using the primer pairs 5'-TACA TACATA**G**TTAAGTTCCTATGTG (sense) and 5'- TAGGAACTTAA**C**TATGTATGTAG (antisense) (former *Afl*II site underlined, mutated base in bold) and 5'-TGGGTGCTT*C**G**ATGG*CGGTGGTG (sense) and 5'- ACCACCG*CCAT**C**G*AAGC ACCCAG (antisense) (former *Nco*I site in italic, mutated base in bold). The Venus (5) DNA sequence was amplified using the sense primer 5'-TGGTGCCATGGCTGT GAGCAAGGGCGAGG and the antisense primer 5'-ATTATA*CTTAAG*C**TTA**CTTGTA CAGCTCG (*Nco*I site underlined, *Afl*II site in italics, stop codon in bold) and introduced into the *Nco*I and *Afl*II sites located 3' of the Sin1^{Part2} DNA sequence in pPSin1-Sin1^{Part1}-mT2^{N}-Sin1^{Part2}-TSin1/fcpNAT, thereby generating the plasmid pPSin1-Sin1^{Part1}-mT2^{N}-Sin1^{Part2}-Venus^{C}-TSin1/fcpNAT. To restore the original *Afl*II and *Nco*I sites in the promoter region, the PSin1-Sin1^{Part1} sequence in this plasmid was then replaced with the equivalent sequence of plasmid pPSin1-Sin1^{Part1}-GFP^{N}-Sin1^{Part2}-TSin1/fcpNAT (see above) using the *Apa*I and *Kpn*I sites flanking this region. In this plasmid Sin1 is expressed with a N-terminal mT2-tag and a C-terminal Venus-tag (Sin1-mT2^{N}-GFP^{C}) under control of the endogenous Sin1 regulatory sequences. Transformation of *T. pseudonana* and selection of transformants on nourseothricin containing agar plates was performed as described previously (3).

Antibody accessibility experiments. The binding of anti-rSin1 and anti-GFP antibodies to biosilica and organic matrices from Sin1-GFP^{N} expressing *T. pseudonana* was quantified as follows. Biosilica was isolated by SDS/EDTA extraction as described previously (2), and fragmented by sonication with a MS72 sonotrode tip (Bandelin) by applying a total energy of 1.612 kJ over 80 s. The insoluble organic matrix material was prepared by incubating the biosilica with 10 M NH_4F (adjusted to pH 4.5 with HCl) for 1 h at room temperature, followed by washing twice with H_2O

through centrifugation (10 min, 10,000 xg) and resuspension. The biosilica and the insoluble organic matrix were resuspended separately in blocking solution (Roti-ImmunoBlock (Carl Roth) supplemented with 0.05% (v/v) Tween 20 (Merck Millipore)) and immobilized on poly-L-lysine-coated coverslips by incubation for 1 hour at room temperature. Unbound material was removed from the coverslips by washing with blocking solution. The coverslips were overlaid with anti-rSin1 antiserum or preimmune serum (each serum was used at 1:1000 dilution) in blocking solution for 1 h. After washing 4x 5 min in TBS (50 mM Tris-HCl pH 7.5, 150 mM NaCl) containing 0.05% (v/v) Tween 20, the coverslips were overlaid with Alexafluor647-conjugated anti-rabbit IgG antibodies (0.67 µg mL^{-1}) in blocking solution for 1 hour. The coverslips were washed as described above, followed by two 5 min washes with TBS. GFP- and Alexafluor647-bearing samples were visualized using epi-illumination with a 488-nm laser and a 647-nm laser at 10 mW and 1 mW, respectively, and respective filter sets (laser bandpass (475/35, 628/40), dichroic longpass (H 488 LPXR, H 643 LPXR), and emission bandpass (525/45, 700/75). The recorded fluorescence intensities were adjusted to prevent saturation of the detector. GFP and Alexafluor647 z-stage image series were subsequently acquired with the NIS-Elementssoftware (Nikon) using an EM CCD camera (Ixon Ultra 897, Andor) mounted on an inverted fluorescence microscope (NSTORM, Nikon) equipped with a 100 oil objective (CFI TIRF Apochromat, numerical aperture 1.49, WD 0.12 mm, Nikon) and an autofocus system (Nikon) at an exposure time of 300 ms (GFP) and 70 ms (Alexafluor647) and 4 frames/µm. For the defined regions of interest, maximum projections displaying the highest value of each pixel in all frames of the z-stacks were created using the software NIS-Elements. Alexafluor647 fluorescence intensity values were normalized by dividing through the corresponding GFP fluorescence intensity values from the same region of interest, resulting in relative fluorescence intensities (RFIs). The degree of accessibility of organic microrings in the biosilica was calculated through dividing the RFI from biosilica by the RFI from microrings.

Immunodetection of GFP in biosilica and organic microrings. Biosilica and organic microrings were isolated from wild-type cells and from transformant cells expressing Sin1-GFP^{N} and Sin1-GFP^{C} using a published protocol (2). Immunolabeling was performed as described under “Antibody accessibility experiments” (see above) using a polyclonal anti-GFP antibody (Clontech; final concentration: 15 µg ml^{-1}) as primary antibody, and an Alexafluor647-conjugated anti-rabbit IgG (Thermo Fisher Scientific; final concentration: 2 µg ml^{-1}) as secondary antibody. Fluorescence imaging (GFP, Alexafluor647) was performed as described above.

Supporting Figures

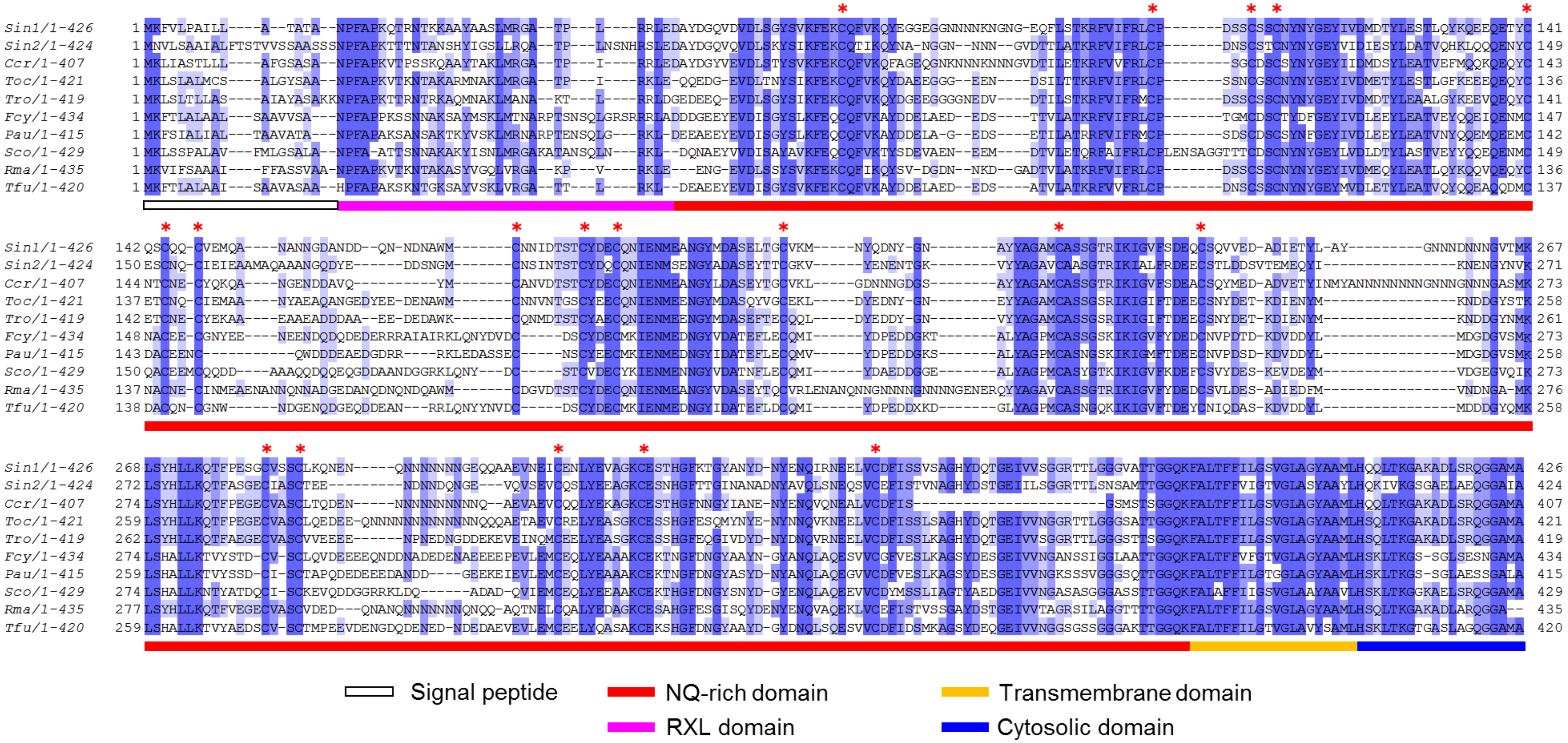

Fig. S1. Sequence conservation of silicanins in diatoms and other protists. Sequences were aligned using ClustalW (6). The alignment was adjusted manually to highlight the conservation of cysteine residues (red asterisks). The degree of sequence identity among all sequences is indicated by the intensity of the background color. A residue with a dark blue background is conserved in all species whereas a lighter blue background indicates that the residue is conserved only in a subset of species. Organisms: *Cyclotella cryptica* (Ccr), *Thalassiosira oceanica* (Toc), *Thalassiosira rotula* (Tro), *Fragilariopsis cylindrus* (Fcy), *Pseudo-nitzschia australis* (Pau), *Staurosira complex* (Sco), *Rhizochromulina marina* (Rma), *Tiarina fusa* (Tfu).

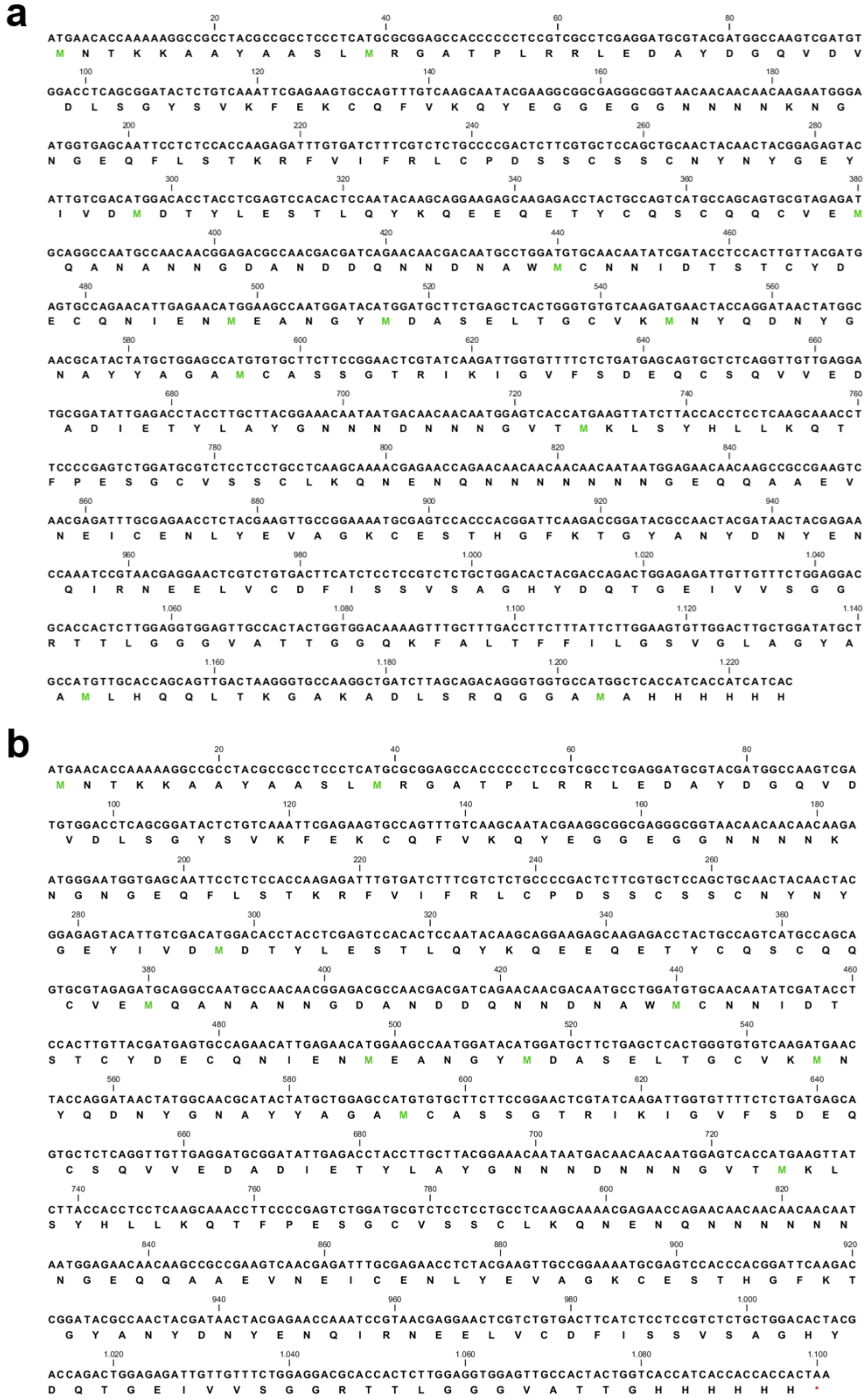

Fig. S2. DNA and amino acid sequences of **(*a*)** rSin1^{-SP} and **(*b*)** rSin1^{lum}.

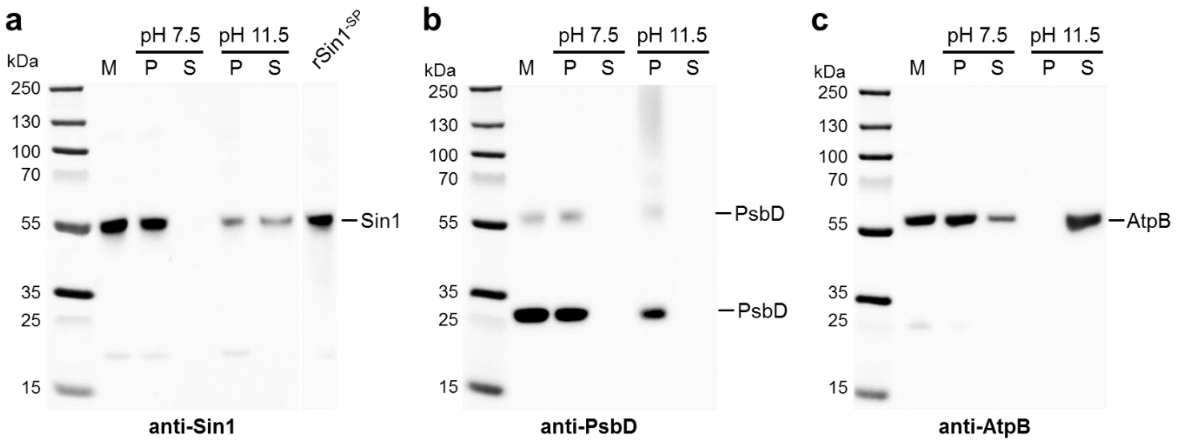

Fig. S3. Membrane association of Sin1. Total membranes from *T. pseudonana* were extracted with lysis buffer (pH 7.5) or carbonate buffer (pH 11.5). Membrane suspensions were incubated on ice and centrifuged (100,000 xg, 1 hour). Pellet (P), supernatant (S), and total membranes before extraction (M) were probed for the presence of **(*a*)** Sin1, **(*b*)** PsbD (integral transmembrane protein with 5 transmembrane helices), and **(*c*)** AtpB (peripheral transmembrane protein) by Western blot analysis with polyclonal antibodies. The faint band between 15-25 kDa in the M and P lanes of **(*a*)** could either be a degradation product of Sin1 or an unrelated protein that incidentally cross-reacts with the anti-Sin1 antibody.

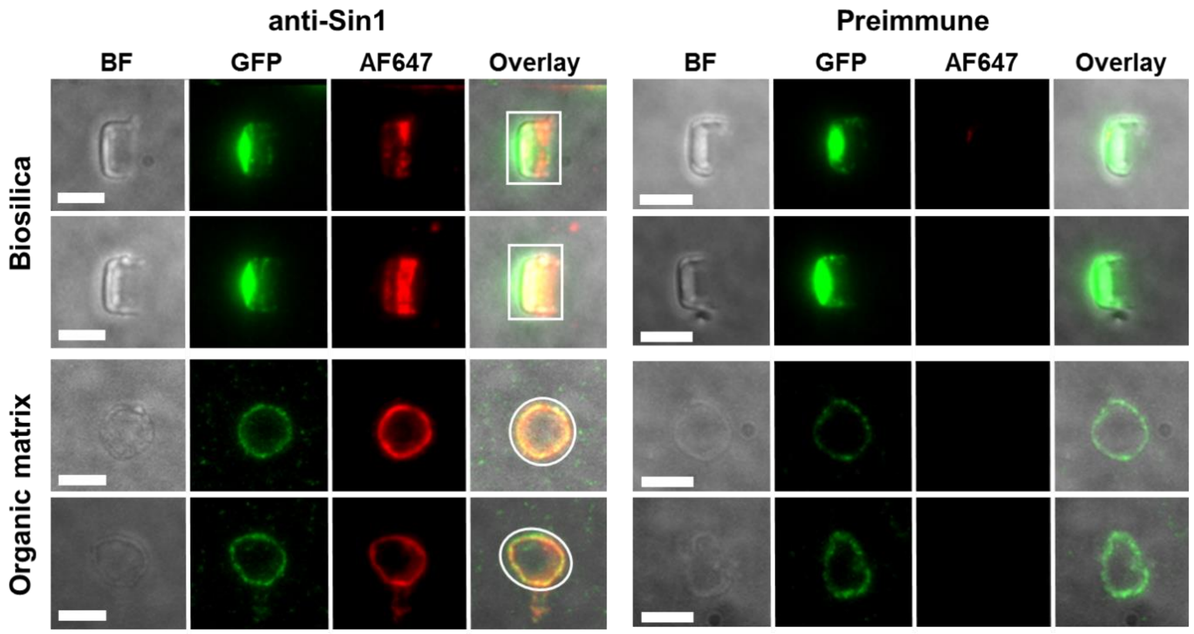

Fig. S4. Accessibility of Sin1 in biosilica and organic matrices. The experiment was conducted according to a previously published method (2). Biosilica and silica-free, insoluble organic matrices were isolated from a transformant strain expressing Sin1-GFP^{N}, and subjected to immunolabeling using anti-rSin1 as primary antibody and an Alexafluor647-labeled secondary antibody. GFP and Alexafluor647 fluorescence was quantified in individual biosilica and organic matrix particles. As a control the same experiment was performed with preimmune serum as the primary antibody. The BF column shows bright field microscopy images and the GFP and AF647 columns show corresponding epifluorescence microscopy images of the same objects in the GFP and Alexafluor647 channels, respectively. The Overlay column shows the overlays of the images from the other three columns. The white rectangles and circles show the regions of interest in which the GFP and Alexafluor647 fluorescence intensities were quantified. Scale bars: 5 µm.

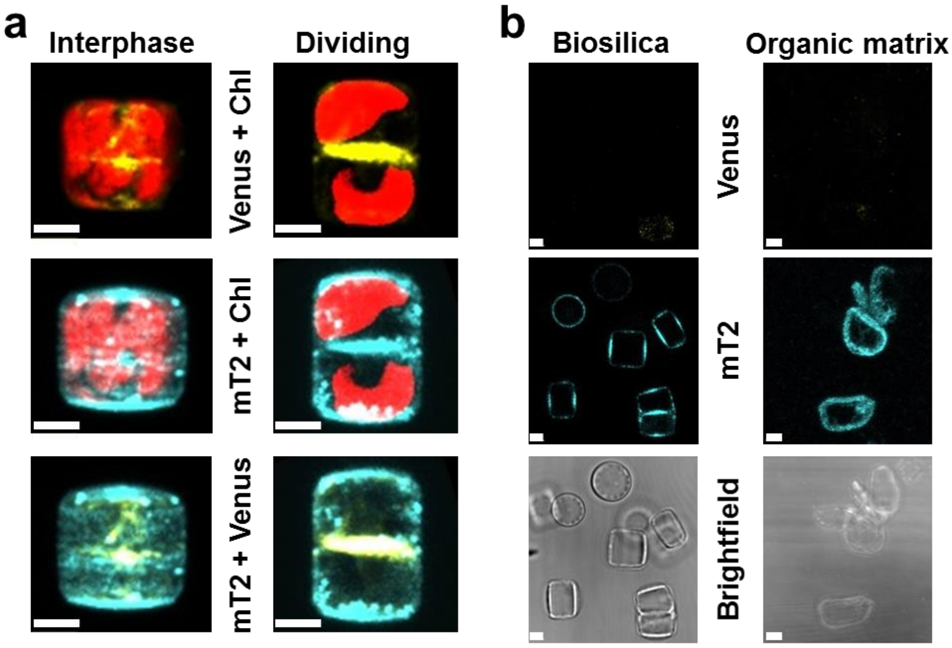

Fig. S5. Localization of a double-tagged Sin1 fusion protein (Sin1-mT2^{N}-Venus^{C}) in *T. pseudonana*. Live cells, biosilica, and biosilica-associated organic matrix from the transformant strain expressing Sin1-mT2^{N}-Venus^{C} (mT2 = mTurquoise2 inserted between the RXL domain and the NQ-rich domain; Venus fused to the C-terminus of Sin1). The fusion protein was expressed under control of the endogenous Sin1 promoter and terminator sequence. Magenta indicates the fluorescence of mTurquoise2 and yellow the fluorescence of Venus. The panels in **(*a*)** show confocal fluorescence images (all z-stacks) of identical individual cells in girdle view during interphase and cell division in the Venus channel (top), the mT2 channel (middle), and the overlay of both channels (bottom). For orientation chlorophyll autofluorescence (red color) is shown in the top and middle row, but was omitted in the overlay images. **(*b*)** Isolated biosilica particles (central z-stack) and organic matrix material (all z-stacks) were imaged by confocal fluorescence microscopy in the Venus channel (top) and the mT2 channel (middle), and by brightfield microscopy (bottom). Scale bars for all images: 2 µm.

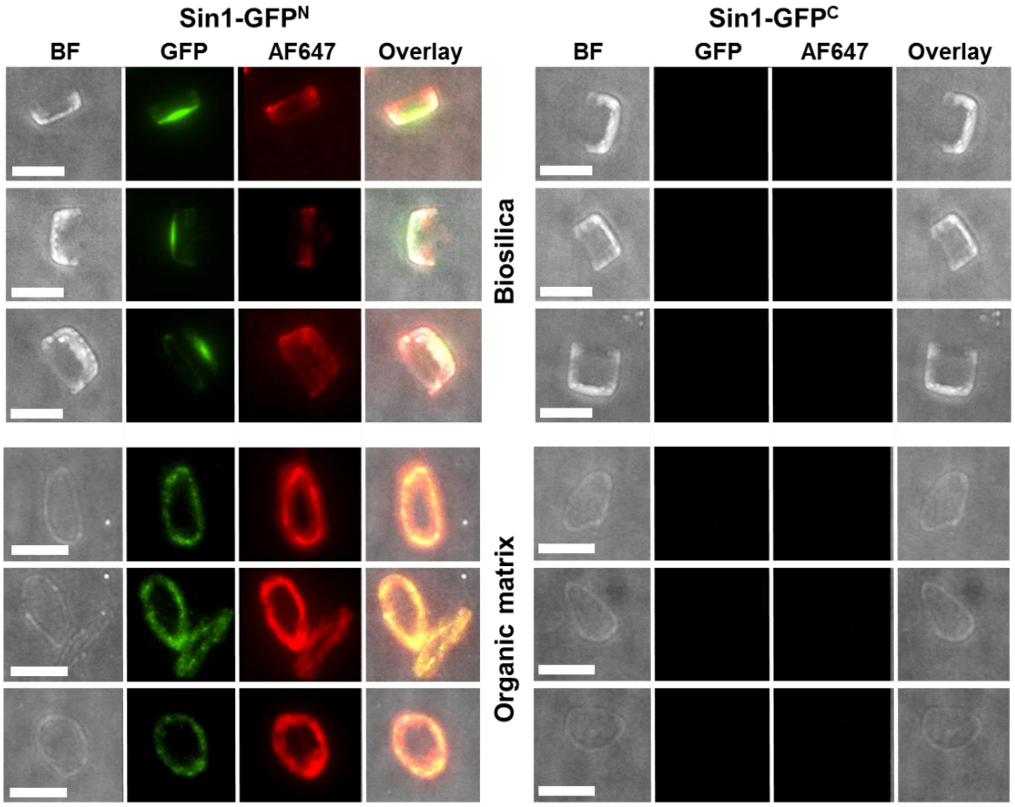

Fig. S6. Anti-GFP immunolabeling of biosilica and organic matrices isolated from *T. pseudonana* transformant cells expressing Sin1-GFP^{C} or Sin1-GFP^{N}. Biosilica and silica-free, insoluble organic matrices were subjected to immunolabeling using anti-GFP as primary antibody and an Alexafluor647-labeled secondary antibody. The BF column shows bright field microscopy images. The GFP and AF647 columns show corresponding epifluorescence microscopy images of the same objects in the GFP and Alexafluor647 channels, respectively. The overlay column shows the overlays of the images from the other three columns. Scale bars: 5 µm.

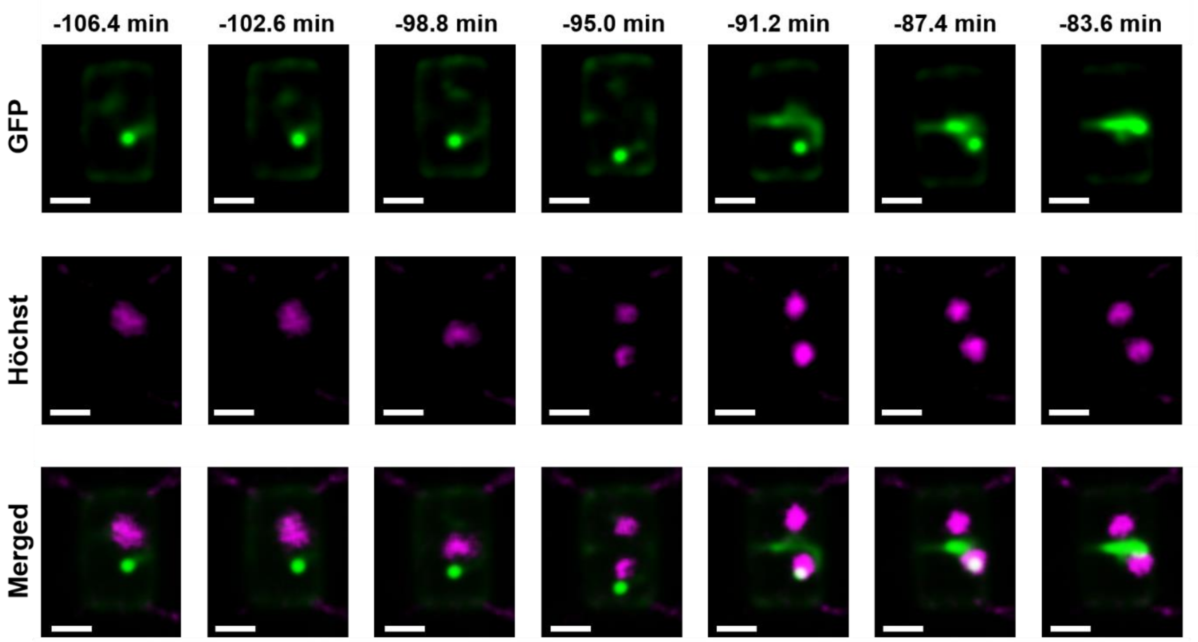

Fig. S7. Time-lapse imaging of a live cell around the time of nuclear division. Selected images from time-lapse confocal fluorescence microscopy of a Sin1-GFP^{C} expressing cells stained with the DNA binding dye Hoechst 34580 are shown (see Movie S2). The time above the images relates to the peak of the GFP fluorescence just prior to valve exocytosis which is set as t = 0 min. GFP fluorescence is shown in green and Hoechst 34580 fluorescence in magenta. The images demonstrate that nuclear division was completed between -98.8 min and -95.0 min before the onset of valve SDV biogenesis between -87.4 and -83.6 min. This time frame is in good agreement with observations on Sin1-GFP^{C} expressing cells that were labeled with PDMPO (see Fig. 4). All images are z-projections of all planes. Scale bars: 2 µm.

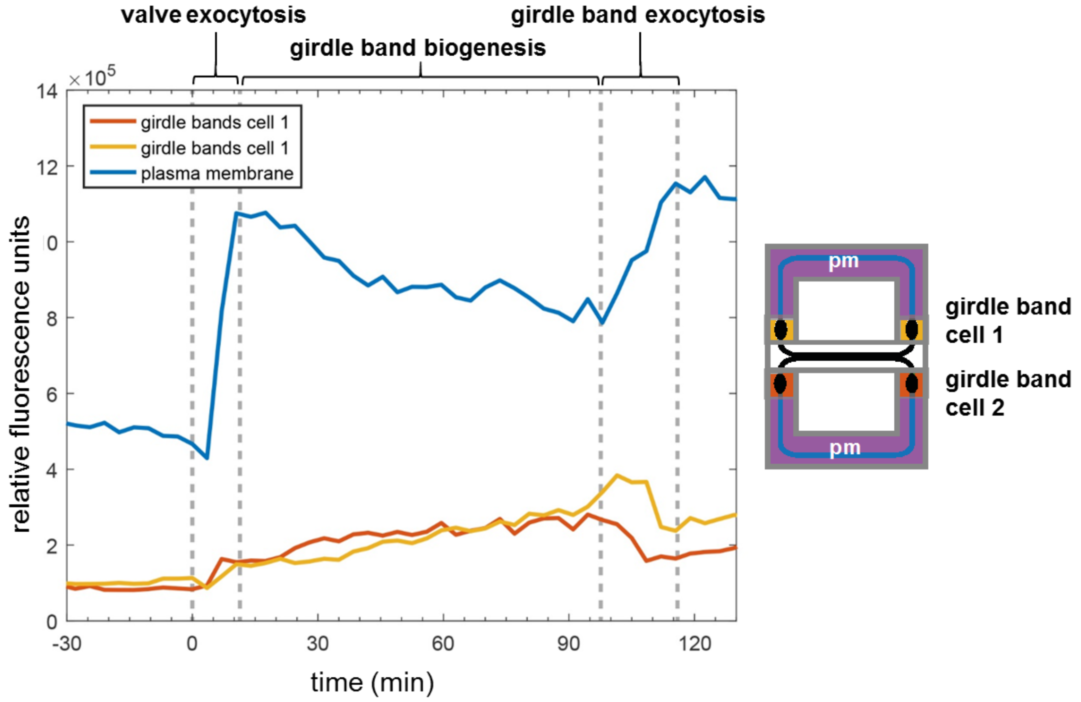

Fig. S8. Time-dependent quantitative analysis of region-specific GFP fluorescence during girdle band formation in a Sin1-GFP^{C} expressing cell. Images were recorded in 3.5 min intervals, and the fluorescence intensities in different regions of the cell were determined. From each frame z-projections were generated combining all nine z-planes. The schematic shows the delineations of the cellular regions that were analyzed. The line coloring in the graphs corresponds to the coloring of the cellular regions in the schematic. The frame with maximum GFP fluorescence in the mid-cell region (trace not shown) was defined as t = 0 min.

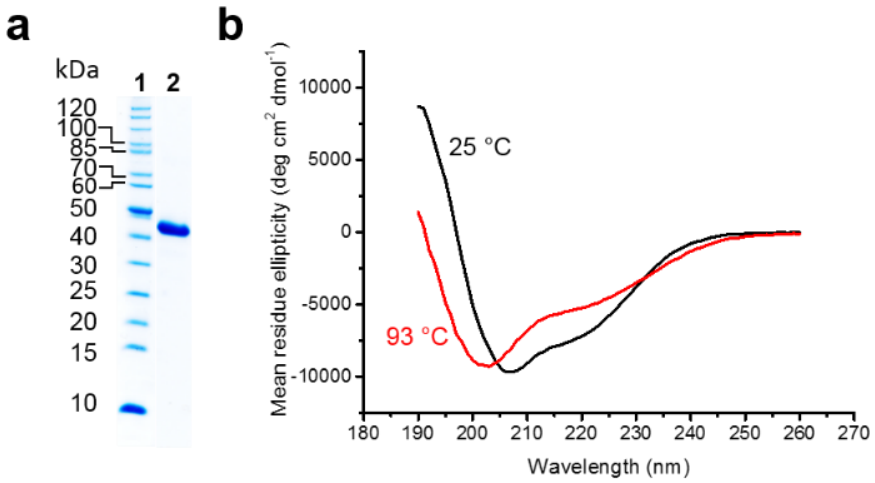

Fig. S9. Biochemical analysis of rSin1^{lum}. **(*a*)** Coomassie stained SDS-PAGE loaded with molecular mass standard proteins (lane 1) and 3 µg purified rSin1^{lum} (lane 2). **(*b*)** Circular dichroism spectra of rSin1^{lum} in 10 mM sodium phosphate buffer pH 7.7 at 25 °C (black line) and at 93 °C (red line).

5'-end of Sin1

```
  1 GGGGGGGGGGGGGGGGGAACACCAACTCCACCTGCCATAATTCGCTACACACCCCCCAAA
 61 TACACCACCAAACGCAGCACCATGAAGTTCGTACTCCCCGCAATCCTCCTCGCCACCGCC
121 ACGGCCAACCCCTTCGCCCCCAAGCAAACCCGCAACACCAAAAAGGCCGCCTACGCCGCC
181 TCCCTCATGCGCGGAGCCACCCCCCTCCGTCGCCTCGAGGATGCGTACGAT
```

3'-end of Sin1

```
  1 ATATGCTGCCATGTTGCACCAGCAGTTGACTAAGGGTGCCAAGGCTGATCTTAGCAGACA
 61 GGGTGGTGCCATGGCTTAAGTCTCGTTTGAAGATTAAGCCATTGTCTTGGTGTGCTGTGA
121 CGTGAGAGAGGATGGAGGGAGTGTCGTAGTCTCTCTAGAATGGTGTGTTGTGGATAAAAG
181 CAAGCACATCATGCTGTATGTTTTGAAGCCTAAGATATAATTTTGTGATTAGTCATTAAA
241 AAAAAAAAAAAAAAAAA
```

Fig. S10. DNA sequences obtained from 5'- and 3'-RACE PCR analysis of Sin1 mRNA. Start and stop codons are highlighted in red, polyG- and polyA-tails are underlined.

Supporting Tables

Table S1. Sin1 homologues identified through tBLASTn searches. The data were grouped into non-diatoms, centric diatoms (i.e. cells with radial symmetry), and pennate diatoms (i.e. cells with bilateral symmetry). Multiple entries for the same species result from redundancies in the database (e.g. one species sequenced multiple times, multiple assemblies for one species).

**Non-diatom**			
**MMETSP entry**	**BLAST hit**	**BLAST E-value**	**BLAST Score**
Rhizochromulina_marina_CCMP1243_SRR1300381_MMETSP1173	c10071_g1_i1 len=1425 path=[1403:0-1424]	1e-148	434
Tiarina_fusa_LIS_SRR1296771_MMETSP0472_alt_Tiarina_fusus	c9607_g1_i1 len=1452 path=[87:0-492 86:493-1451]	3e-115	350
Blepharisma_japonicum_Stock-R1072_SRR1294460_MMETSP1395	c13816_g1_i1 len=517 path=[1:0-516]	2e-46	160
Minchinia_chitonis__SRR1300215_MMETSP0186	c716_g1_i1 len=329 path=[1:0-328]	9e-29	104
Lessardia_elongata_SPMC-104_SRR1300468_MMETSP1147	c406_g1_i1 len=210 path=[1:0-209]	2e-21	84.0
Madagascaria_erythrocladioides_CCMP3234_SRR1300528_MMETSP1450	c23020_g1_i1 len=349 path=[327:0-348]	2e-21	89.7
Oxyrrhis_marina_LB1974_SRR1300474_MMETSP1426	c49188_g1_i1 len=229 path=[207:0-228]	7e-20	85.5
Aureococcus_anophagefferens_CCMP1850_SRR1300280_MMETSP0916	c20414_g1_i1	2e-19	82.4
uncultured_eukaryote_CCMP2293_SRR1300243_MMETSP0989	c31666_g1_i1 len=230 path=[208:0-229]	6e-19	82.4
uncultured_eukaryote_CCMP2293_SRR1300241_MMETSP0986	c53680_g1_i1 len=204 path=[1:0-203]	1e-18	81.3
uncultured_eukaryote_CCMP2293_SRR1300242_MMETSP0988	c56015_g1_i1 len=222 path=[1:0-221]	1e-18	81.3
Prasinoderma_coloniale_CCMP1413_SRR1296862_MMETSP0806_2	c6180_g1_i1 len=261 path=[1:0-260]	8e-15	69.7
Synchroma_pusillum_CCMP3072_SRR1300531_MMETSP1452	c7938_g1_i1 len=215 path=[1:0-214]	1e-13	65.9
Durinskia_baltica_CSIRO-CS-38_SRR1296839_MMETSP0116_2	c103628_g1_i1 len=437 path=[1:0-436]	6e-11	62.0
uncultured_eukaryote_CCMP2135_SRR1300533_MMETSP1467	c8734_g1_i1 len=207 path=[1:0-206]	1e-10	57.8
Durinskia_baltica_CSIRO-CS-38_SRR1296941_MMETSP0117_2	c5595_g1_i1 len=1389 path=[137:0-15 @136@!:16-897 1033:898-925 ...	6e-10	61.6
uncultured_eukaryote_CCMP2111_SRR1300525_MMETSP1446	c8608_g1_i1 len=282 path=[1:0-281]	0.080	31.6
Emiliania_huxleyi_379_SRR1300232_MMETSP0997	c25492_g1_i1 len=305 path=[283:0-304]	0.081	32.7
Emiliania_huxleyi_379_SRR1300230_MMETSP0995	c2469_g1_i2 len=227 path=[1180:0-12 976:13-226]	0.084	32.0

**Pennate diatoms**			
**MMETSP entry**	**BLAST hit**	**BLAST E-value**	**BLAST Score**
Pseudo-nitzschia_australis_10249-10-AB_SRR1296848_MMETSP0140_2	c5425_g1_i1 len=1494 path=[1:0-1493]	5e-109	333
Pseudo-nitzschia_australis_10249-10-AB_SRR1296849_MMETSP0141_2	c19344_g1_i1 len=1492 path=[1:0-1491]	5e-109	333
Pseudo-nitzschia_australis_10249-10-AB_SRR1296847_MMETSP0139_2	c15567_g1_i1 len=1505 path=[1:0-1504]	6e-109	333
Pseudo-nitzschia_australis_10249-10-AB_SRR1296850_MMETSP0142_2	c3053_g1_i1 len=1511 path=[1480:0-1479 3003:1480-1510]	6e-109	333
Thalassionema_frauenfeldii_CCMP-1798_SRR1296708_MMETSP0786	c4228_g1_i1 len=1363 path=[1365:0-1362]	4e-108	330

Staurosira_CCMP2646_SRR1300449_MMETSP1361	c3498_g1_i1 len=1479 path=[1:0-1478]	1e-107	330
Pseudo-nitzschia_heimii_UNC1101_SRR1300452_MMETSP1423	c12539_g1_i1 len=1613 path=[1591:0-1612]	6e-106	327
Thalassionema_nitzschioides_L26-B_SRR1296923_MMETSP0158	c20510_g1_i1 len=1470 path=[1448:0-1469]	2e-105	324
Thalassionema_nitzschioides_L26-B_SRR1296922_MMETSP0156	c23985_g1_i1 len=1534 path=[1:0-1533]	4e-105	324
Pseudo-nitzschia_fraudulenta_WWA7_SRR1294395_MMETSP0852	c7920_g1_i1 len=1470 path=[1:0-1469]	8e-104	320
Pseudo-nitzschia_fraudulenta_WWA7_SRR1296745_MMETSP0850	c9023_g1_i1 len=1534 path=[1512:0-1533]	1e-103	320
Pseudo-nitzschia_fraudulenta_WWA7_SRR1296747_MMETSP0853	c12755_g1_i1 len=1524 path=[1:0-1523]	1e-103	320
Pseudo-nitzschia_fraudulenta_WWA7_SRR1296746_MMETSP0851	c13654_g1_i1 len=1530 path=[1:0-1529]	4e-103	320
Tryblionella_compressa_CCMP561_SRR1296829_MMETSP0744_alt_Nitzschia_punctata	c8101_g1_i1 len=1556 path=[1768:0-898 2664:899-919 240:920-1555]	4e-103	319
Tryblionella_compressa_CCMP561_SRR1296830_MMETSP0745_alt_Nitzschia_punctata	c4801_g1_i1 len=1583 path=[1561:0-925 2487:926-946 2508:947-1582]	4e-103	319
Tryblionella_compressa_CCMP561_SRR1296832_MMETSP0747_alt_Nitzschia_punctata	c4841_g1_i1 len=1575 path=[1693:0-648 2339:649-669 98:670-1574]	4e-103	319
Tryblionella_compressa_CCMP561_SRR1296831_MMETSP0746_alt_Nitzschia_punctata	c6786_g1_i1 len=1590 path=[1:0-919 921:920-940 942:941-972 974:...	5e-103	319
Cyclophora_tenuis_ECT3854_SRR1296719_MMETSP0397	c7879_g1_i1 len=2065 path=[53:0-2064]	1e-102	322
Stauroneis_constricta_CCMP1120_SRR1300408_MMETSP1352	c5024_g1_i1 len=1558 path=[3163:0-541 268:542-617 @344@!:618-97...	2e-102	317
Amphora_coffeiformis_CCMP127_SRR1296713_MMETSP0316	c5384_g1_i1 len=1282 path=[1:0-1281]	3e-102	313
Amphora_coffeiformis_CCMP127_SRR1296713_MMETSP0316	c11081_g1_i1 len=1308 path=[1286:0-1307]	5e-102	313
Amphora_coffeiformis_CCMP127_SRR1296713_MMETSP0316	c15517_g1_i1 len=1329 path=[1307:0-1328]	8e-102	313
Pseudo-nitzschia_pungens_cf.-cingulata_SRR1300393_MMETSP1060	c979_g1_i1 len=1557 path=[1585:0-1556]	1e-100	313
Asterionellopsis_glacialis_CCMP1581_SRR1300451_MMETSP1394	c4365_g1_i1 len=1606 path=[2126:0-611 2737:612-631 505:632-659 ...	4e-100	311
Thalassiothrix_antarctica_L6-D1_SRR1296920_MMETSP0152	c6096_g1_i1 len=1453 path=[83:0-80 164:81-123 189:124-154 220:1...	8e-100	309
Striatella_unipunctata_CCMP2910_SRR1296706_MMETSP0800	c11594_g1_i3 len=1480 path=[4975:0-145 6769:146-210 5183:211-25...	4e-99	308
Fragilariopsis_kerguelensis_L2-C3_SRR1296820_MMETSP0909	c27603_g1_i1 len=1547 path=[1:0-1546]	3e-97	304
Fragilariopsis_kerguelensis_L2-C3_SRR1296819_MMETSP0906	c14276_g1_i1 len=1606 path=[1:0-1605]	5e-97	304
Fragilariopsis_kerguelensis_L2-C5_SRR1296912_MMETSP0736	c3453_g1_i1 len=1603 path=[1631:0-1602]	8e-97	303
Fragilariopsis_kerguelensis_L2-C5_SRR1296910_MMETSP0734	c6008_g1_i1 len=1636 path=[1:0-1635]	1e-96	303
Fragilariopsis_kerguelensis_L2-C5_SRR1296909_MMETSP0733	c6598_g1_i1 len=1692 path=[1670:0-1691]	2e-96	303
Fragilariopsis_kerguelensis_L2-C3_SRR1296967_MMETSP0908	c7654_g1_i1 len=1713 path=[1691:0-1712]	3e-96	303
Nitzschia_RCC80_SRR1294404_MMETSP0014_2	c13380_g1_i2 len=1956 path=[1934:0-254 2189:255-390 2325:391-58...	2e-95	304
Attheya_septentrionalis_CCMP2084_SRR1300527_MMETSP1449	c18253_g1_i1 len=1760 path=[1738:0-1759]	3e-95	300
Amphiprora_paludosa_CCMP125_SRR1300396_MMETSP1065	c9423_g1_i1 len=1734 path=[1712:0-1733]	3e-94	298
Pseudo-nitzschia_arenysensis_B593_SRR1296720_MMETSP0329	c8221_g1_i1 len=1600 path=[1744:0-1523 3262:1524-1599]	5e-94	296
Eucampia_antarctica_CCMP1452_SRR1300514_MMETSP1437	c4037_g1_i1 len=1491 path=[1469:0-1490]	3e-92	290
Cylindrotheca_closterium_KMMCC-B-181_SRR1294406_MMETSP0017_2	c8162_g1_i1 len=1586 path=[1630:0-924 2554:925-933 @34@!:934-14...	2e-91	290

Asterionellopsis_glacialis__SRR1296963_MMETSP0713	c6964_g1_i1 len=1414 path=[1:0-1413]	3e-91	286
Asterionellopsis_glacialis_CCMP134_SRR1296937_MMETSP0705	c2758_g1_i1 len=1386 path=[1364:0-577 1942:578-1385]	6e-90	282
Asterionellopsis_glacialis_CCMP134_SRR1296808_MMETSP0706	c14966_g1_i1 len=1366 path=[1:0-1365]	7e-90	282
Asterionellopsis_glacialis_CCMP134_SRR1296977_MMETSP0707	c17542_g1_i1 len=1379 path=[1:0-1378]	1e-89	282
Amphiprora_CCMP467_SRR1296826_MMETSP0725	c15192_g1_i1 len=1416 path=[1:0-1415]	3e-86	273
Amphiprora_CCMP467_SRR1296826_MMETSP0725	c2301_g1_i1 len=1449 path=[1:0-658 660:659-683 685:684-1448]	4e-86	274
Amphiprora_CCMP467_SRR1296826_MMETSP0725	c14169_g1_i1 len=1511 path=[1489:0-1510]	7e-86	273
Amphiprora_CCMP467_SRR1296825_MMETSP0724	c3819_g1_i1 len=1541 path=[1:0-726 3156:727-751 753:752-1540]	2e-85	273
Entomoneis_sp._CCMP2396_SRR1300522_MMETSP1443	c2622_g1_i1 len=1591 path=[1691:0-1525 3214:1526-1537 89:1538-1...	2e-84	270
Grammatophora_oceanica_CCMP-410_SRR1296957_MMETSP0009_2	c29705_g1_i1 len=985 path=[1:0-984]	6e-71	229
Craspedostauros_australis_CCMP3328_SRR1300521_MMETSP1442	c1458_g1_i1 len=915 path=[893:0-856 1750:857-914]	1e-66	217
Pseudo-nitzschia_delicatissima_B596_SRR1296721_MMETSP0327	c4529_g2_i1 len=991 path=[1:0-237 5098:238-990]	3e-62	207
Pseudo-nitzschia_delicatissima_UNC1205_SRR1300464_MMETSP1432	c17668_g1_i1 len=986 path=[1:0-985]	1e-61	205
Asterionellopsis_glacialis_CCMP134_SRR1296809_MMETSP0708	c488_g1_i1 len=876 path=[1:0-875]	5e-57	191
Fragilariopsis_kerguelensis_L2-C5_SRR1296911_MMETSP0735	c9821_g1_i1 len=1041 path=[1:0-1040]	5e-50	175
Fragilariopsis cylindrus	JGI CCMPM02:Project 16035:Fracy1_assembly_scaffolds.fasta.gz	2e-88	

**Centric diatoms**			
**MMETSP entry**	**BLAST hit**	**BLAST E-value**	**BLAST Score**
Thalassiosira_FW_SRR1300392_MMETSP1059	c670_g1_i1 len=1529 path=[1507:0-1528]	0.0	676
Thalassiosira_rotula_GSO102_SRR1296988_MMETSP0910	c5549_g1_i1 len=1504 path=[1790:0-566 2357:567-591 2382:592-640...	5e-168	485
Thalassiosira_gravida_GMp14c1_SRR1296986_MMETSP0493	c4626_g1_i1 len=1462 path=[1440:0-1001 2442:1002-1461]	2e-167	483
Thalassiosira_rotula_GSO102_SRR1296989_MMETSP0911	c5827_g1_i2 len=1459 path=[1864:0-176 @144@!:177-669 2533:670-9...	2e-166	479
Cyclotella_meneghiniana_CCMP-338_SRR1300390_MMETSP1057	c9676_g1_i1 len=1587 path=[523:0-272 795:273-288 88:289-381 900...	1e-162	473
Thalassiosira_minuscula_CCMP1093_SRR1296956_MMETSP0740	c6880_g1_i1 len=1458 path=[255:0-23 279:24-195 450:196-204 459:...	2e-162	469
Thalassiosira_rotula_CCMP3096_SRR1296877_MMETSP0404_2	c10827_g1_i2 len=1585 path=[4777:0-518 5295:519-542 568:543-777...	3e-162	471
Thalassiosira_minuscula_CCMP1093_SRR1296953_MMETSP0737	c13590_g1_i1 len=1478 path=[269:0-70 340:71-609 120:610-611 122...	8e-159	461
Thalassiosira_antarctica_CCMP982_SRR1296821_MMETSP0904	c16916_g1_i1 len=1436 path=[1:0-1435]	3e-158	459
Thalassiosira_antarctica_CCMP982_SRR1296962_MMETSP0903	c4936_g1_i1 len=1510 path=[1581:0-315 1897:316-339 @1921@!:340-...	3e-158	460
Thalassiosira_rotula_GSO102_SRR1300436_MMETSP0913	c6127_g1_i1 len=1574 path=[1551:0-178 1730:179-309 1861:310-384...	4e-158	459
Thalassiosira_antarctica_CCMP982_SRR1296965_MMETSP0905	c7374_g1_i1 len=1477 path=[2:0-270 273:271-310 313:311-603 606:...	7e-158	459
Thalassiosira_minuscula_CCMP1093_SRR1296954_MMETSP0738	c9912_g1_i1 len=1391 path=[157:0-386 3038:387-432 590:433-704 8...	1e-156	454
Thalassiosira_antarctica_CCMP982_SRR1296959_MMETSP0902	c4083_g1_i1 len=1477 path=[1602:0-293 3127:294-317 1923:318-654...	2e-156	455
Thalassiosira_rotula_GSO102_SRR1296990_MMETSP0912	c6080_g1_i1 len=1552 path=[1872:0-466 2338:467-541 @200@!:542-1...	3e-156	455
Detonula_confervacea_CCMP-353_SRR1300391_MMETSP1058	c6268_g1_i1 len=1572 path=[1715:0-54 1770:55-260 104:261-268 @1...	7e-153	447
Thalassiosira_minuscula_CCMP1093_SRR1296955_MMETSP0739	c9223_g1_i2 len=1442 path=[197:0-149 347:150-194 3740:195-203 @...	4e-152	444

Thalassiosira_rotula_CCMP3096_SRR1296755_MMETSP0403	c11264_g1_i1 len=1835 path=[2737:0-12 855:13-577 2:578-601 3325...	4e-151	446
Thalassiosira_NH16_SRR1300274_MMETSP1071	c12826_g1_i1 len=1623 path=[3387:0-11 525:12-87 3472:88-88 @160...	1e-147	434
Thalassiosira_weissflogii_CCMP1010_SRR1294465_MMETSP1411	c12439_g1_i1 len=1637 path=[1615:0-1636]	1e-147	434
Thalassiosira_weissflogii_CCMP1010_SRR1296880_MMETSP0900_2	c10375_g1_i1 len=1619 path=[1:0-1618]	1e-147	434
Thalassiosira_weissflogii_CCMP1010_SRR1296881_MMETSP0901_2	c8755_g1_i1 len=1634 path=[1664:0-385 2050:386-1633]	1e-147	434
Thalassiosira_weissflogii_CCMP1010_SRR1300477_MMETSP1406	c20482_g1_i1 len=1643 path=[1:0-1642]	1e-147	434
Thalassiosira_weissflogii_CCMP1010_SRR1300478_MMETSP1407	c12720_g1_i1 len=1637 path=[1:0-1636]	1e-147	434
Thalassiosira_weissflogii_CCMP1010_SRR1300479_MMETSP1408	c1927_g1_i1 len=1618 path=[1:0-1617]	1e-147	434
Thalassiosira_weissflogii_CCMP1010_SRR1300480_MMETSP1409	c12305_g1_i1 len=1634 path=[1:0-1633]	1e-147	434
Thalassiosira_weissflogii_CCMP1010_SRR1300481_MMETSP1410	c15370_g1_i1 len=1620 path=[1598:0-1619]	1e-147	434
Thalassiosira_weissflogii_CCMP1010_SRR1300486_MMETSP1416	c2291_g1_i1 len=1639 path=[1:0-1611 3256:1612-1638]	1e-147	434
Thalassiosira_weissflogii_CCMP1010_SRR1300490_MMETSP1420	c17283_g1_i1 len=1639 path=[1:0-1638]	1e-147	434
Thalassiosira_weissflogii_CCMP1010_SRR1296879_MMETSP0899_2	c10420_g1_i1 len=1656 path=[1634:0-1655]	2e-147	434
Thalassiosira_weissflogii_CCMP1010_SRR1300476_MMETSP1405	c15474_g1_i1 len=1652 path=[1:0-1651]	2e-147	434
Thalassiosira_weissflogii_CCMP1010_SRR1300482_MMETSP1412	c20390_g1_i1 len=1639 path=[1617:0-1638]	2e-147	434
Thalassiosira_weissflogii_CCMP1010_SRR1300485_MMETSP1415	c11879_g1_i1 len=1656 path=[1634:0-1655]	2e-147	434
Thalassiosira_weissflogii_CCMP1010_SRR1300487_MMETSP1417	c1307_g1_i1 len=1658 path=[1:0-1631 3297:1632-1657]	2e-147	434
Thalassiosira_weissflogii_CCMP1010_SRR1300488_MMETSP1418	c12210_g1_i1 len=1639 path=[1:0-1638]	2e-147	434
Thalassiosira_weissflogii_CCMP1010_SRR1300491_MMETSP1422	c20429_g1_i1 len=1646 path=[1624:0-1645]	2e-147	434
Thalassiosira_weissflogii_CCMP1336_SRR1296931_MMETSP0878	c19888_g1_i1 len=1663 path=[1641:0-1662]	2e-147	434
Thalassiosira_weissflogii_CCMP1336_SRR1296932_MMETSP0879	TR5313-c0_g1_i1 len=1641 path=[1619:0-1640] [-1, 1619, -2]	2e-147	434
Thalassiosira_weissflogii_CCMP1336_SRR1296933_MMETSP0880	c14442_g1_i1 len=1646 path=[1:0-1645]	2e-147	434
Thalassiosira_weissflogii_CCMP1336_SRR1296934_MMETSP0881	c17309_g1_i1 len=1677 path=[1:0-1676]	2e-147	434
Thalassiosira_weissflogii_CCMP1010_SRR1300489_MMETSP1419	c17354_g1_i1 len=1686 path=[1664:0-1685]	3e-147	434
Thalassiosira_punctigera_Tpunct2005C2_SRR1300398_MMETSP1067	c12626_g1_i4 len=1613 path=[1:0-137 7420:138-144 146:145-178 18...	1e-140	417
Thalassiosira_gravida_GMp14c1_SRR1296987_MMETSP0494	c6828_g1_i1 len=1094 path=[1072:0-1093]	7e-131	385
Skeletonema_menzellii_CCMP793_SRR1296801_MMETSP0604	c6649_g1_i1 len=1549 path=[1725:0-1548]	4e-130	388
Skeletonema_menzellii_CCMP793_SRR1296800_MMETSP0603	c4528_g1_i1 len=1546 path=[89:0-1545]	5e-130	388
Skeletonema_marinoi_UNC1201_SRR1300462_MMETSP1428	c6290_g1_i1 len=1525 path=[1505:0-36 1542:37-56 @1562@!:57-1333...	2e-128	384
Skeletonema_grethae_CCMP-1804_SRR1296994_MMETSP0578_alt_Skeletonema_grethea	c7696_g1_i1 len=1635 path=[191:0-185 377:186-253 445:254-637 36...	5e-128	384
Skeletonema_japonicum_CCMP2506_SRR1296995_MMETSP0593	c8543_g1_i1 len=1782 path=[613:0-868 1475:869-884 261:885-1060 ...	5e-126	380
Skeletonema_marinoi_skelA_SRR1300407_MMETSP0918	c7636_g1_i1 len=1489 path=[1729:0-1328 3055:1329-1359 75:1360-1...	1e-125	376
Skeletonema_marinoi_skelA_SRR1300409_MMETSP0920	c8151_g1_i1 len=1508 path=[169:0-158 328:159-184 142:185-1507]	1e-125	376
Skeletonema_dohrnii_SkelB_SRR1296855_MMETSP0563	c8438_g1_i2 len=1496 path=[2870:0-970 3832:971-1331 1315:1332-1...	7e-125	374
Skeletonema_dohrnii_SkelB_SRR1296795_MMETSP0562	c8746_g1_i1 len=1532 path=[1498:0-921 2420:922-924 2423:925-969...	9e-125	374

Skeletonema_marinoi_FE60_SRR1300260_MMETSP1040.Trinity	c4957_g1_i1 len=1524 path=[1952:0-222 2174:223-244 53:245-456 2...	1e-124	374
Skeletonema_costatum_1716_SRR1294403_MMETSP0013_2	c8199_g1_i1 len=1502 path=[90:0-25 @116@!:26-335 3893:336-339 4...	2e-124	374
Skeletonema_marinoi_SM1012Den-03_SRR1296717_MMETSP0320	c8956_g2_i1 len=1518 path=[5032:0-975 2982:976-999 3006:1000-13...	3e-124	373
Skeletonema_marinoi_FE7_SRR1300259_MMETSP1039	c21484_g1_i1 len=1498 path=[1:0-1497]	3e-123	370
Skeletonema_marinoi_SM1012Hels-07_SRR1296716_MMETSP0319	c9661_g1_i1 len=2900 path=[6147:0-22 1641:23-480 25:481-490 35:...	1e-119	374
Dactyliosolen_fragilissimus__SRR1296964_MMETSP0580	c4745_g1_i1 len=1656 path=[1:0-1655]	4e-111	340
Synedropsis_cf._recta_CCMP1620_SRR1300382_MMETSP1176_alt_Synedropsis_recta_cf	c26877_g1_i1 len=1442 path=[1420:0-1441]	6e-111	338
Thalassiosira_weissflogii_CCMP1010_SRR1300483_MMETSP1413	c15798_g1_i1 len=1128 path=[1106:0-1127]	4e-110	332
Licmophora_paradoxa_CCMP2313_SRR1300448_MMETSP1360	c9183_g1_i1 len=1488 path=[105:0-1487]	7e-110	336
Thalassiosira_oceanica_CCMP1005_SRR1300227_MMETSP0972	c5857_g1_i1 len=992 path=[1020:0-991]	7e-110	330
Thalassiosira_oceanica_CCMP1005_SRR1300226_MMETSP0971	c6409_g1_i1 len=990 path=[1076:0-580 1656:581-585 82:586-989]	8e-110	330
Thalassiosira_weissflogii_CCMP1010_SRR1294466_MMETSP1421	c16187_g1_i1 len=1218 path=[1196:0-1217]	9e-110	332
Thalassiosira_oceanica_CCMP1005_SRR1300225_MMETSP0970	c26648_g1_i1 len=999 path=[1:0-998]	1e-109	330
Thalassiosira_weissflogii_CCMP1010_SRR1296878_MMETSP0898_2	c2963_g1_i1 len=1221 path=[1199:0-1220]	1e-109	332
Thalassiosira_weissflogii_CCMP1010_SRR1300484_MMETSP1414	c5118_g1_i1 len=1446 path=[1424:0-839 2264:840-1445]	6e-109	333
Thalassiosira_oceanica_CCMP1005_SRR1294424_MMETSP0973	c8068_g1_i1 len=1401 path=[2521:0-1400]	9e-109	332
Chaetoceros_neogracile_CCMP1317_SRR1296836_MMETSP0754	c6870_g1_i2 len=1558 path=[1702:0-535 2238:536-536 @2239@!:537-...	1e-108	333
Thalassiosira_gravida_GMp14c1_SRR1296992_MMETSP0492	c18166_g1_i1 len=904 path=[1:0-903]	1e-108	325
Chaetoceros_neogracile_CCMP1317_SRR1296835_MMETSP0753	c5265_g1_i1 len=1555 path=[53:0-1554]	5e-108	332
Chaetoceros_neogracile_CCMP1317_SRR1296834_MMETSP0752	c6349_g1_i2 len=1547 path=[61:0-16 @78@!:17-506 568:507-528 590...	6e-108	332
Chaetoceros_neogracile_CCMP1317_SRR1296833_MMETSP0751	c266_g1_i1 len=1628 path=[1656:0-1627]	2e-107	331
Chaetoceros_sp._UNC1202_SRR1300463_MMETSP1429	c6725_g1_i1 len=1495 path=[1:0-1494]	1e-106	327
Astrosyne_radiata_13vi08-1A_SRR1296718_MMETSP0418	c5904_g1_i1 len=1547 path=[83:0-377 67:378-1546]	4e-103	318
Chaetoceros_cf._neogracile_RCC1993_SRR1300373_MMETSP1336	c4325_g1_i1 len=1474 path=[149:0-1473]	7e-103	317
Chaetoceros_affinis_CCMP159_SRR1294453_MMETSP0088	c2123_g1_i1 len=1489 path=[1:0-1488]	1e-100	310
Chaetoceros_affinis_CCMP159_SRR1294451_MMETSP0088	c252_g1_i1 len=1559 path=[1:0-650 3117:651-1558]	2e-100	310
Chaetoceros_affinis_CCMP159_SRR1294446_MMETSP0088	c1716_g1_i1 len=1597 path=[1:0-1596]	3e-100	310
Chaetoceros_affinis_CCMP159_SRR1294447_MMETSP0088	c3567_g1_i1 len=1604 path=[1:0-1603]	3e-100	310
Chaetoceros_affinis_CCMP159_SRR1294448_MMETSP0088	c1726_g1_i1 len=1597 path=[1:0-1596]	3e-100	310
Chaetoceros_affinis_CCMP159_SRR1294450_MMETSP0088	c7398_g1_i1 len=1590 path=[1568:0-1589]	3e-100	310
Chaetoceros_affinis_CCMP159_SRR1294452_MMETSP0088	c2095_g1_i1 len=1592 path=[1:0-1591]	3e-100	310
Thalassiothrix_antarctica_L6-D1_SRR1296921_MMETSP0154	c6354_g1_i1 len=1462 path=[1621:0-1461]	1e-99	309
Chaetoceros_affinis_CCMP159_SRR1294449_MMETSP0088	c9622_g1_i2 len=1652 path=[1756:0-30 3924:31-36 1793:37-143 190...	2e-99	310
Chaetoceros_affinis_CCMP159_SRR1294474_MMETSP0088	c4630_g1_i1 len=1613 path=[57:0-1062 1120:1063-1173 1230:1174-1...	2e-99	310
Chaetoceros_affinis_CCMP159_SRR1294475_MMETSP0088	c622_g1_i1 len=1724 path=[1:0-1279 1281:1280-1282 1284:1283-1723]	4e-99	310

Chaetoceros_curvisetus__SRR1294454_MMETSP0717	c7326_g2_i1 len=1310 path=[105:0-1268 1372:1269-1309]	8e-99	305
Chaetoceros_debilis_MM31A-1_SRR1296918_MMETSP0149	c6292_g1_i1 len=1734 path=[1922:0-366 2288:367-373 64:374-1733]	1e-96	304
Chaetoceros_debilis_MM31A-1_SRR1296919_MMETSP0150	c8125_g1_i1 len=1745 path=[753:0-181 934:182-291 630:292-507 12...	3e-96	303
Coscinodiscus_wailesii_CCMP2513_SRR1300397_MMETSP1066	c11619_g2_i1 len=1492 path=[4187:0-49 4062:50-73 4086:74-97 411...	7e-95	297
Ditylum_brightwellii_GSO104_SRR1300435_MMETSP1012	c20397_g2_i1 len=1490 path=[6943:0-321 8927:322-356 374:357-399...	9e-94	295
Odontella_sinensis_Grunow-1884_SRR1296882_MMETSP0160_2	c6693_g1_i1 len=1555 path=[1:0-520 3136:521-525 527:526-1554]	8e-91	287
Stephanopyxis_turris_CCMP-815_SRR1296867_MMETSP0794_2	c3171_g1_i1 len=1450 path=[455:0-709 1163:710-725 @1179@!:726-1...	6e-88	279
Odontella_aurita_isolate-1302-5_SRR1294405_MMETSP0015_2	c23993_g1_i4 len=1543 path=[4621:0-125 6738:126-167 4798:168-19...	2e-87	279
Extubocellulus_spinifer_CCMP396_SRR1296805_MMETSP0697	c9041_g1_i1 len=1614 path=[217:0-1333 25:1334-1334 26:1335-1415...	1e-83	269
Extubocellulus_spinifer_CCMP396_SRR1296806_MMETSP0698	c9544_g1_i1 len=1587 path=[1796:0-981 119:982-1000 @138@!:1001-...	1e-83	269
Extubocellulus_spinifer_CCMP396_SRR1296807_MMETSP0699	c6243_g1_i1 len=1594 path=[1580:0-1414 3003:1415-1447 3036:1448...	1e-83	269
Ditylum_brightwellii_GSO105_SRR1300291_MMETSP0998	c2165_g1_i1 len=1337 path=[1342:0-156 14:157-1336]	9e-83	264
Proboscia_inermis_CCAP1064-1_SRR1296824_MMETSP0816	c24702_g1_i1 len=1511 path=[1:0-1510]	2e-82	265
Rhizosolenia_setigera_CCMP-1694_SRR1296707_MMETSP0789	c7288_g1_i2 len=1481 path=[2632:0-1026 319:1027-1480]	3e-82	264
Proboscia_alata_PI-D3_SRR1296927_MMETSP0176	c8177_g1_i1 len=1514 path=[1592:0-1513]	9e-82	263
Ditylum_brightwellii_Pop1--SS4-_SRR1300272_MMETSP1062	c5442_g1_i1 len=1368 path=[53:0-1367]	1e-81	262
Ditylum_brightwellii_Pop2--SS10-_SRR1300273_MMETSP1063	c6175_g1_i1 len=1358 path=[1386:0-1357]	2e-81	261
Chaetoceros_dichaeta_CCMP1751_SRR1300526_MMETSP1447	c9256_g1_i1 len=1552 path=[115:0-119 @86@!:120-1400 1513:1401-1...	3e-80	259
Leptocylindrus_danicus_B650_SRR1296723_MMETSP0321	c3479_g1_i1 len=1572 path=[1887:0-84 338:85-1571]	5e-80	259
Chaetoceros_brevis_CCMP164_SRR1300510_MMETSP1435	c2717_g1_i2 len=1362 path=[545:0-103 14:104-176 86:177-1155 158...	1e-79	253
Leptocylindrus_danicus_CCMP1856_SRR1300450_MMETSP1362	c5071_g1_i1 len=1534 path=[1:0-1533]	5e-79	256
Corethron_pennatum_L29A3_SRR1296925_MMETSP0171	c10390_g1_i3 len=1370 path=[4343:0-12 73:13-43 104:44-64 125:65...	6e-76	247
Ditylum_brightwellii_GSO104_SRR1300293_MMETSP1010	c15898_g1_i1 len=1212 path=[1:0-1211]	5e-73	237
Leptocylindrus_aporus_B651_SRR1296722_MMETSP0322_alt_Leptocylindrus_danicus_var._apora	c13594_g1_i1 len=1526 path=[1504:0-1525]	2e-71	236
Corethron_hystrix_308_SRR1294401_MMETSP0010_2	c5841_g1_i2 len=1699 path=[3179:0-150 65:151-1698]	7e-71	236
Ditylum_brightwellii_GSO105_SRR1300292_MMETSP1001	c6946_g1_i1 len=1181 path=[1159:0-1180]	2e-70	230
Ditylum_brightwellii_GSO104_SRR1300294_MMETSP1013	c6838_g1_i2 len=1394 path=[447:0-60 @508@!:61-1236 3272:1237-12...	2e-69	229
Proboscia_alata_PI-D3_SRR1296926_MMETSP0174	c8780_g1_i1 len=1204 path=[1182:0-1203]	2e-69	228
Ditylum_brightwellii_GSO103_SRR1294415_MMETSP1002	c682_g1_i1 len=1230 path=[61:0-118 179:119-123 30:124-1229]	6e-68	221
Triceratium_dubium_CCMP147_SRR1300492_MMETSP1175	c8777_g1_i1 len=1071 path=[835:0-230 1066:231-247 1083:248-305 ...	1e-64	214
Ditylum_brightwellii_GSO103_SRR1294414_MMETSP1002	c12381_g1_i1 len=924 path=[902:0-542 3637:543-543 3638:544-557 ...	2e-60	201
Chaetoceros_sp._GSL56_SRR1296891_MMETSP0200_2	c11321_g1_i1 len=957 path=[935:0-956]	3e-60	201
Chaetoceros_affinis_CCMP159_SRR1294445_MMETSP0088	c10628_g1_i3 len=988 path=[899:0-86 3965:87-88 986:89-372 1268:...	3e-55	188
Aulacoseira_subarctica_CCAP-1002-5_SRR1300395_MMETSP1064	c21181_g1_i1 len=1460 path=[1:0-1459]	1e-48	174
Cyclotella cryptica		1e-96	

Table S2. Sequence identities between *T. pseudonana* (Tps) Sin1 and Sin2, and between homologues from other diatoms and non-diatom organisms. *Cyclotella cryptica* (Ccr), *Thalassiosira oceanica* (Toc), *Thalassiosira rotula* (Tro); ii) pennate diatoms: *Fragilariopsis cylindrus* (Fcy), *Pseudo-nitzschia australis* (Pau), *Staurosira complex* (Sco); iii) non-diatom organisms *Rhizochromulina marina* (Rma), *Tiarina fusa* (Tfu).

	**Centric diatoms**				**Pennate diatoms**			**Non-diatoms**	
**Organism**	**Tps**	**Ccr**	**Toc**	**Tro**	**Fcy**	**Pau**	**Sco**	**Rma**	**Tfu**
**AA number**	424	407	421	419	434	415	429	435	420
**Sequence identity (%)**	55	66	66	66	47	46	46	62	49

Table S3. Quantification of the extractability of Sin1, PsbD and AtpB from *T. pseudonana* membranes. Western blots of the respective experiments (see Fig. S2) were analyzed using the software Image Lab 5.2.1 (Biorad). The exposure times were chosen to ensure that chemiluminescence intensities of all protein bands were (i.e. not saturated within the dynamic range of the camera. Data of three independent experiments were averaged. The membrane extractability (E) in % was calculated according to the following equation

$$\mathrm{E} = \frac{\mathrm{I_S}}{\mathrm{I_S}+\mathrm{I_P}} \times 100.$$

in which I_S is the intensity of the protein band in the extract and I_P the intensity of the protein band in the extracted membrane.

Extraction buffer	Sin1	AtpB	PsbD
	E (%)	E (%)	E (%)
Lysis buffer pH 7.5	0	39 ± 10	0
Carbonate buffer pH 11.5	56 ± 3	97 ± 4	1 ± 0

Table S4. Quantification of the accessibility of Sin1 in biosilica. The experiment was conducted according to a previously published method (2). RFI = relative fluorescence intensity (the standard error of the mean is provided), n = number of particles analyzed. Biosilica and insoluble organic matrices were isolated from *T. pseudonana* cells expressing Sin1-GFP^N, and subjected to immunolabeling using anti-Sin1 as primary antibody and an Alexafluor647-labeled secondary antibody. Fluorescence intensities were determined using epifluorescence microscopy (see Fig. S3). Alexafluor647 fluorescence intensity in individual biosilica particles served as a quantitative measure for accessibility of Sin1 for the antibody. The same immunolabeling experiment was performed with the organic matrix and the resulting Alexafluor647 fluorescence intensity served as reference value for maximum microring accessibility under the assay conditions. For each analyzed individual object (i.e. biosilica particle, organic matrix particle) the ratio of the fluorescence intensities (RFI) of Alexafluor647 and GFP was calculated thereby normalizing the immunolabeling intensity to the amount of antigen that was present in each object. Therefore, the ratio of the RFI for biosilica (RFI_{BS}) and the RFI for the organic matrix (RFI_{MR}) indicates the fraction of biosilica-associated Sin1 that is accessible to the antibody molecules.

Sample		RFI	RFI_{BS} / RFI_{OM}
**BS**	Girdleband biosilica	6.66 ±0.85 (n=33)	0.19
	Valve biosilica	1.21 ±0.11 (n=43)	0.03
**OM**	Organic matrices	35.19 ±3.28 (n=36)	n. a.

Table S5. Secondary structure analysis of rSin1^{lum}. CD spectra (see Fig. S9b) were analysed using the DiChroWeb software (7, 8) and the CDSSTR method (reference data set 7 from reference 7), and compared to the amino acid sequence-based secondary structure predicted by the PsiPred webserver (8).

	Temperature (°C)	α helix (%)	β sheet (%)	Turns (%)	Disordered (%)
Predicted	-	28	14	-	58
Experiment	25	18	22	18	43
	93	9	16	13	62

Table S6. Determination of free sulfhydryl groups in rSin1^{lum}. The concentration of free thiol groups [SH] at three different protein concentrations [rSin1^{lum}] was determined using cysteine as a standard. The ratio of free thiol groups that were detected per rSin1^{lum} molecule was less than 1, which indicated that in the vast majority of rSin1^{lum} molecules all 18 Cysteine residues were engaged in disulfide bonds.

[rSin1^{lum}] (µM)	[SH] detected (µM)	[SH] : [rSin1^{lum}]
2.78	0.9	0.32
5.56	1.8	0.32
8.33	3.1	0.37

Supporting Movies

proj2_4way_blue.avi

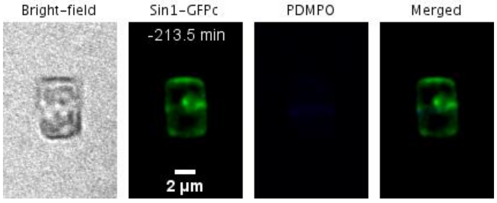

Movie S1. Time-lapse imaging of a Sin1-GFP^{C} expressing live cell around the time of valve biogenesis. Bright-field (left), GFP fluorescence (second from left), and PDMPO fluorescence (third from left) images were acquired in 3.5 min intervals. Movies showing GFP fluorescence, PDMPO fluorescence, and an overlay of both (right) are z-projections of all planes. The bright-field images represent the central z plane. The indicated times relate to the maximum of PDMPO fluorescence during valve biogenesis (t = 0 min).

proj3_4way_new.avi

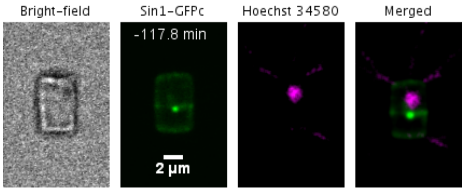

Movie S2. Time-lapse imaging of a live-cell around the time of nuclear division. Bright-field (left), GFP fluorescence (second from left), and Hoechst 34580 fluorescence (third from left) images were acquired in 3.8 min intervals. Movies showing GFP fluorescence, Hoechst 34580 fluorescence, and an overlay of both (right) are z-projections of all planes. The bright-field images represent the central z plane. The indicated times relate to the maximum of GFP fluorescence during valve biogenesis (t = 0 min).

proj4_4way_blue.avi

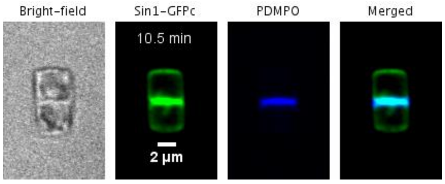

Movie S3. Time-lapse imaging of a live cell around the time of girdle band biogenesis. Bright-field (left), GFP fluorescence (second from left), and PDMPO fluorescence (third from left) images were acquired in 3.5 min intervals. Movies showing GFP fluorescence, PDMPO fluorescence, and an overlay of both (right) are z-projections of all planes. The bright-field images represent the central z plane. The indicated times relate to the maximum of PDMPO fluorescence during valve biogenesis (t = 0 min).

References

1. Poulsen N., Kröger N. Silica morphogenesis by alternative processing of silaffins in the diatom *Thalassiosira pseudonana*. *J Biol Chem* **279**, 42993–42999 (2004).
2. Kotzsch A., Pawolski D., Milentyev A., Shevchenko A., Scheffel A., Poulsen N., Shevchenko A., Kröger N. Biochemical composition and assembly of biosilica-associated insoluble organic matrices from the diatom *Thalassiosira pseudonana*. *J Biol Chem* **291**, 4982-4997 (2016).
3. Poulsen N., Chesley P.M., Kröger N. Molecular genetic manipulation of the diatom *Thalassiosira pseudonana* (Bacillariophyceae). *J Phycol* **42**, 1059–1065 (2006).
4. Goedhart J., von Stetten D., Noirclerc-Savoye M., Lelimousin M., Joosen L., Hink M.A., van Weeren L., Gadella Jr. T.W.J., Royant A. (2012) Structure-guided evolution of cyan fluorescent proteins towards a quantum yield of 93%. *Nat. Commun.* **3**, 751.
5. Nagai T., Ibata K., Park E.S., Kubota M., Mikoshiba K., Miyawaki A. (2002) A variant of yellow fluorescent protein with fast and efficient maturation for cell-biological applications. *Nat. Biotechnol.* **20**, 87-90.
6. Thompson J.D., Higgins D.G., Gibson T.J. CLUSTAL W: improving the sensitivity of progressive multiple sequence alignment through sequence weighting, position specific gap penalties and weight matrix choice. *Nucleic Acids Res* **22**, 4673-4680 (1994).
7. Sreerama N., Woody R.W. Estimation of protein secondary structure from CD spectra: Comparison of CONTIN, SELCON and CDSSTR methods with an expanded reference set. *Anal Biochem* **287**, 252-260 (2000).
8. Jones D.T. Protein secondary structure prediction based on position-specific scoring matrices. *J Mol Biol* **292**,195-202 (1999).
